# User-Driven Development of a Digital Behavioral Intervention for Chronic Pain: Multimethod Multiphase Study

**DOI:** 10.2196/74064

**Published:** 2025-07-08

**Authors:** Afra Selma Taygar, Sara Laureen Bartels, Rocío de la Vega, Ida Flink, Linnéa Engman, Suzanne Petersson, Sophie I Johnsson, Katja Boersma, Lance M McCracken, Rikard K Wicksell

**Affiliations:** 1 Department of Clinical Neuroscience Karolinska Institutet Stockholm Sweden; 2 Department of Psychiatry and Neuropsychology Maastricht University Maastricht The Netherlands; 3 Department of Personality, Assessment and Psychological Treatment Universidad de Málaga Málaga Spain; 4 Instituto de Investigación Biomédica de Málaga Málaga Spain; 5 Department of Social and Psychological Studies Karlstad University Karlstad Sweden; 6 Pain clinic Saint Göran Hospital Stockholm Sweden; 7 School of Behavioural, Social and Legal Sciences Center for Health and Medical Psychology (CHAMP) Örebro University Örebro Sweden; 8 Department of Medicine and Optometry Linnaeus University Kalmar Sweden; 9 Department of Molecular Medicine and Surgery Karolinska Institutet Stockholm Sweden; 10 Department of Clinical Psychology Uppsala University Uppsala Sweden

**Keywords:** chronic pain, digital therapeutics, behavioral intervention, development, end-user involvement

## Abstract

**Background:**

Recent research shows that chronic pain affects 27% of the adult population. For many, pain significantly impairs quality of life and everyday functioning. Behavioral interventions have shown utility, but access remains limited. Digital health solutions can increase reach, but there is a need for user-friendly, feasible, and evidence-based digital interventions.

**Objective:**

This study aimed to clarify how a digital behavioral intervention for people with chronic pain can be developed through a user-centered approach to address the needs and preferences of the target population.

**Methods:**

This study used a multimethod approach involving end users, namely, patients with chronic pain and therapists, to develop prototypes for a digital behavioral intervention across 3 phases. In the preparation phase (phase 0), fictional patient personas (n=3) were created to represent the diversity of the target population while emphasizing transdiagnostic features across people with chronic pain. In the design phase (phase 1), qualitative data from focus groups with patients (n=5; aged 37-51 years; 4/5, 80% women; 2/5, 40% diagnosed with Ehlers-Danlos syndrome; 3/5, 60% either undiagnosed or uncertain about their diagnosis) and therapists (n=12 licensed psychologists; aged 29-64 years; 9/12, 75% women) were collected to explore end-user preferences for the intervention design and content. In the testing phase (phase 2), the initial full prototype of the digital intervention was piloted with patients (n=11; aged 36-58 years; 9/11, 82% women; with diverse diagnoses, including migraine, arthritis, fibromyalgia, complex regional pain syndrome, hypermobile Ehlers-Danlos syndrome, herniated disc, chronic fatigue syndrome, and 1/11, 9% cases of undiagnosed pain) and therapists (n=3 licensed psychologists; aged 36-58 y; 3/3, 100% women). The Consolidated Framework for Implementation Research was used to structure analyses of end-user feedback.

**Results:**

On the basis of end-user input, a 6-week digital behavioral intervention for chronic pain was created. Focus groups highlighted the importance of accessibility and adaptability of the digital intervention, emphasizing the need for tailored content, flexibility (eg, contact with the therapist via asynchronous messaging, telephone, or video calls), and user-friendly design (eg, easy navigation between modules, short microsessions, and visualizations). Average weekly ratings (scale from 1=not at all to 7=very much) by patients during pilot-testing indicated that the intervention was helpful (mean range 4.27-5.45, SD range 1.20-2.20), enjoyable (mean range 3.81-4.81, SD range 1.12-2.08), and understandable (mean range 4.45-6, SD range 1.30-1.86), suggesting initial acceptability and usability of the intervention.

**Conclusions:**

The results illustrated the utility of the patient personas when preparing, of the focus groups when designing, and of the end-user feedback when testing this new digital intervention for people with chronic pain. The findings indicated that the intervention is promising while also providing relevant end-user suggestions (eg, video content, text-to-speech function, and add-on modules) to guide further improvements.

## Introduction

### Background

Chronic pain affects approximately 27% of adults [1], significantly impacting their daily life and general well-being [2]. Behavioral interventions building on, for instance, the fear-avoidance [3] and psychological flexibility models [4,5], aim to enhance resilience to pain and distress. These interventions have robust empirical support and are increasingly used [6-8]. However, access to evidence-based behavioral interventions remains low [9].

The evolution of digital solutions represents a paradigm shift, with the potential to maximize the accessibility of behavioral treatments, yet there is a scarcity of evidence-based digital treatments in regular health care [10]. To facilitate further development in the field, the innovation process should be concise and efficient [11]. It should be executed within an established *framework* to facilitate standardization, *described* in detail for transparency and quality assurance, and *evaluated* scientifically for data-driven decisions on how to proceed for further testing and implementation [12].

Existing research suggests that user involvement is essential to match digital health interventions to preferences and needs [13-16]. In addition, stakeholders should be consulted early on to facilitate successful and sustainable implementation [17]. Stakeholders include, for instance, *innovation facilitators* such as health care managers and IT developers and *end users* such as patients and health care professionals. In particular, end-user involvement provides insights into relevant needs and priorities [18,19]. Collaboration with end users to tailor the treatment is critical in the development phase to emphasize person-centeredness [20,21]. Furthermore, end-user engagement throughout the development process is essential for creating effective digital health interventions [22] and increasing ease of use [23].

Recently, the UK Medical Research Council recommended that the development of novel interventions involve individuals with lived experiences in each phase (ie, development, feasibility, evaluation, and implementation) to assure inclusivity, accessibility, and efficacy [24]. While an increasing number of studies have identified preferences and needs regarding digital interventions for chronic pain [25-27], end-user involvement is usually limited to a certain phase of the innovation process. How end users can be involved in the implementation and evaluation of digital behavioral interventions has been described [28,29], but clear descriptions of end-user engagement during the development phase remain limited. Thus, there is a need for studies clarifying how the development of novel digital interventions for chronic pain can apply a user-centered approach to facilitate replicability and establish standards for digital health innovation.

### Objectives

Digital Behavioural Health for Chronic Pain (DAHLIA) is a user-centered multiphase project with two distinct yet related purposes: (1) to create an evidence-based digital health intervention for people with chronic pain and (2) to provide a robust and replicable process for user-centered development, evaluation, and implementation [30]. The overarching aim of this study was to clarify how a digital behavioral intervention can be developed through a user-centered approach to address the needs and preferences of the target population. Development included 3 phases: preparation (phase 0), design (phase 1), and testing (phase 2).

More specifically, the research questions were as follows:

1. Phase 0—preparation. How can patient personas be used to define the relevant patient characteristics, needs, and treatment targets of people living with chronic pain? How can a better understanding of patient personas guide the prototype development?
2. Phase 1—design. What are the preferences of end users for the design (ie, content, structure, and format) of this digital intervention as reported in end-user focus groups (patients and therapists)?
3. Phase 2—testing. How do end users (patients and therapists) perceive engaging with the first treatment prototype (version 1.0), and how can the digital intervention be further improved to meet needs and preferences?

## Methods

### Study Design

This study used a multimethod approach in the development of DAHLIA prototypes 1.0 and 2.0 and included 3 phases (0=preparation, 1=design, and 2=testing). This study is part of a larger project also containing evaluation and implementation phases [30], which will be presented elsewhere.

Throughout, *participants* refers to all individuals who took part in the study, including both patients and therapists*.* We used the term *patients* referring to people living with chronic pain and *therapists* referring to people delivering the intervention. Both groups are considered *end users* of the digital intervention, reflecting their involvement in using and evaluating the intervention.

**Figure 1:**
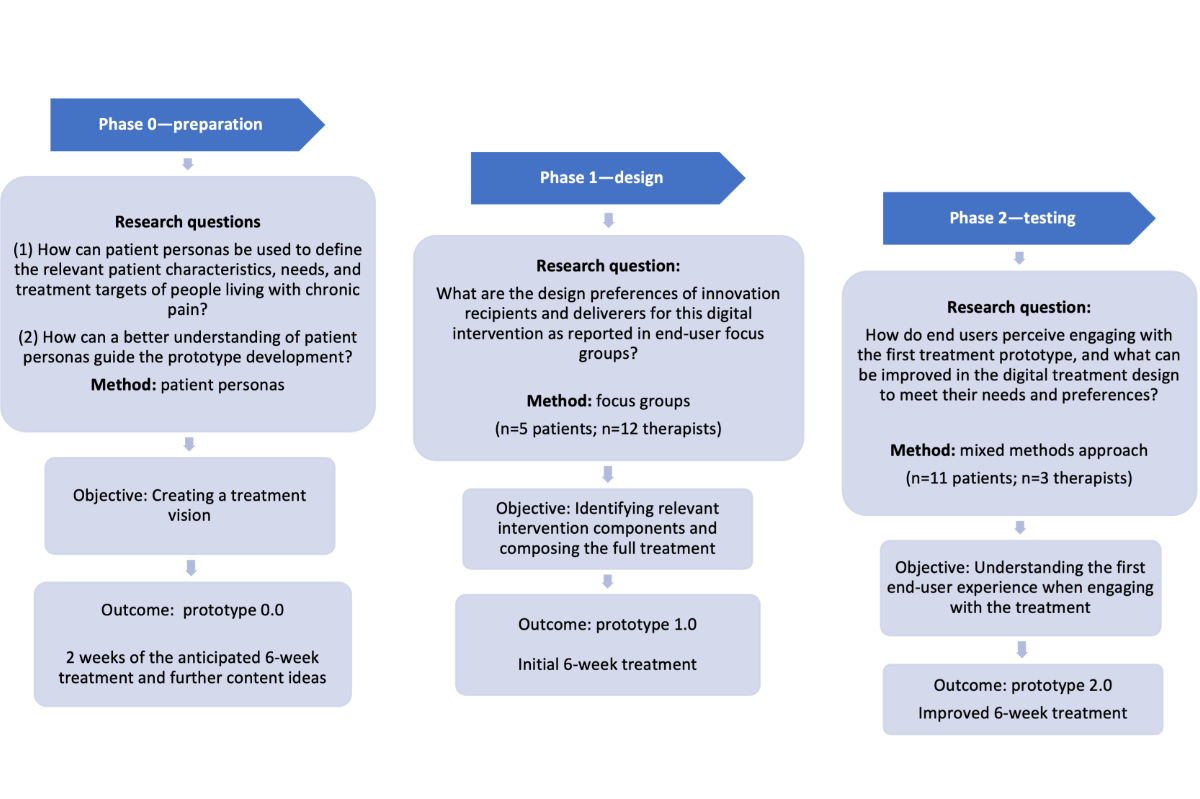
Overview of the development phases (ie, 0=preparation; 1=design; 2=testing) of the DAHLIA digital behavioral intervention for people with chronic pain. The process followed a user-centered design approach involving end users (patients and therapists) throughout.

Intervention development was centered on end users (patients and therapists) and used complementary user engagement approaches, namely, patient personas (phase 0; see the Phase 0 [Preparation]: Preliminary Patient Characteristics, Needs, and Treatment Targets section), qualitative focus groups (phase 1; see the Phase 1 [Design]: User-Centered Design of the Digital Intervention section), and multimethod pilot-testing based on perceived end-user experience (phase 2; see the Phase 2 [Testing]: Piloting the Digital Intervention section). These development phases are presented in Figure 1 and further described in the following sections.

The development of the intervention was reported following the Guidance for the Reporting of Intervention Development (GUIDED) checklist [31]. The intervention was also described using the Template for Intervention Description and Replication checklist [32] (see the completed GUIDED and Template for Intervention Description and Replication checklists in Multimedia Appendix 1 [30-32]), as recommended by the GUIDED checklist.

### Ethical Considerations

This study was approved by the Swedish Ethical Review Authority (approval Dnr 2021-02437) and carried out in accordance with the Declaration of Helsinki. All participants provided informed consent before joining the study, including participation in the focus groups and pilot-testing. Participants also consented to the use of anonymized quotes and data for dissemination. Collected data were stored securely on encrypted servers and were accessible only to the research team. Participants received no compensation.

### Phase 0 (Preparation): Preliminary Patient Characteristics, Needs, and Treatment Targets

To address the first research question, the initial design of the prototype was based on a theoretical framework and conceptual model, as well as a preliminary definition of the patient characteristics, needs, and relevant treatment targets based on patient personas.

#### Theoretical Frameworks and Conceptual Model

The DAHLIA treatment program is based on learning theory [33], with the fear-avoidance model [3] and the psychological flexibility model [4,5] integrated into a comprehensive conceptual model. The primary treatment objective is to increase resilience (ie, being able to sustain living a fulfilling life in the presence of distress) [34] to chronic pain and distress by improving behavioral self-management skills relevant to well-being and functioning [35].

#### Patient Personas

Personas, or fictional user profiles, aim to depict the target group of a treatment or product [36]. As the involvement of people with chronic pain may be challenging in the early stages of development due to ethical or practical reasons, personas can be used as representations of the target group [37]. Personas are a design methodology, and their utility depends on the project aim.

In the DAHLIA project, 3 patient personas were used during the initial conceptualization and preparation of the treatment. Patient personas were used to discuss whether the treatment vision (eg, content, setup, and design) aligned with the needs and characteristics of the target population. The patient personas were designed to represent the heterogeneity (eg, gender, age, and pain conditions) of the target population while emphasizing transdiagnostic features across people living with chronic pain (eg, self-management strategies). The patient personas facilitated an inclusive design process and the identification of common user needs across different patient profiles to guide decisions on the structure and content of the digital intervention.

The patient personas were developed by clinical researchers (RKW, IF, KB, LMC, and SP) inspired by patient personas used in a previous study [38] and edited over several months (SLB and SIJ) until the project partners reached a consensus. The patient personas were informed by existing chronic pain research, particularly the survey of chronic pain in Europe by Breivik et al [39]. Each patient persona consisted of 4 main domains: sociodemographics, pain profile, health care, and personal needs and goals. One of the 3 patient personas used to guide the user-centered design and tailor the intervention content is presented in Multimedia Appendix 2, and the other 2 are presented elsewhere [30,37]. Through narrative synthesis, a summary of the patient persona’s characteristics, key challenges in the development of the intervention, and potential ways to approach these challenges were identified, guiding discussions and decisions to refine the treatment content.

#### Intervention Structure for Prototype Version 0.0

The treatment prototype version 0.0 was prepared based on theoretical considerations and implications of the patient personas following consensus discussions in the project group (see the Results section). Moreover, a microlearning approach [40,41] was applied to organize treatment content into brief and frequent sessions, which was considered useful to prospective users experiencing challenges due to, for instance, dyslexia, fatigue, attention deficits, or reduced cognitive functioning. It was anticipated that the final treatment would include 6 modules, with 4 sessions per module, totaling 24 treatment modalities. Overall, a 6-week treatment duration was anticipated, with 1 module per week. The outcome of phase 0 was treatment prototype version 0.0, including 2 modules of the treatment, building the foundation for end-user input in phase 1.

### Phase 1 (Design): User-Centered Design of the Digital Intervention

Insights from phase 0 (the preparation phase), including the initial understanding of patient needs based on patient personas, informed the development of the prototype in phase 1. The design of the digital health intervention (prototype version 1.0) was based on a preliminary understanding of patient needs and relevant treatment targets and guided by end-user input from focus groups.

#### End-User Focus Groups

To develop the full 6-week treatment, both prospective patients and therapists were involved to provide user input on their needs and preferences regarding the digital intervention. A qualitative study with 2 patient focus groups (n=5 in total) and 3 therapist focus groups (n=12 in total) was conducted. Notably, the initial research plan [30] was to involve 6 to 8 participants per focus group and conduct the discussions face-to-face. However, due to the COVID-19 pandemic, focus groups were conducted digitally, and the target size of each group was reduced to 3 to 5 participants per group based on previous research [42], especially for digital settings [43].

#### Participants and Recruitment

Inclusion criteria to participate in a *patient focus group* were (1) age of 18 to 65 years (working age); (2) pain duration of ≥3 months; (3) ability to communicate in Swedish; and (4) access to a computer, smartphone, and internet connection in a home environment. Individuals were excluded if they had serious psychiatric comorbidities (eg, risk of suicide).

Inclusion criteria for the *therapist focus group* were (1) being a licensed psychologist or psychotherapist with training in cognitive behavioral therapy (CBT), (2) fluency in Swedish, and (3) access to a computer or laptop with an internet connection. Experience treating patients with chronic pain was recommended but not required. No exclusion criteria were specified to increase external validity.

Eligible therapists received an email with a link to provide informed consent. Participants (patients and therapists) were recruited from 2 health care centers in 2 different regions of Sweden (Stockholm and Kalmar). For patient recruitment, flyers were distributed at clinics, and interested individuals scanned a QR code and were directed to a digital system (REDCap [Research Electronic Data Capture; Vanderbilt University] [44,45]) to register. Registered individuals were contacted by a research assistant (SIJ) to receive detailed information about the study. A clinical coordinator (SP, licensed psychologist) screened potential participants to check eligibility.

#### Materials and Procedures

Eligible participants were contacted via email with a link to provide informed consent and sociodemographic information (eg, age, sex, and occupation) in REDCap before the focus group. Therapists also reported their level of experience delivering psychological treatment (number of years), including for people with chronic pain. Patients were asked to complete questions specific to their pain condition.

The focus groups were conducted as 2-hour–long recorded video meetings (Microsoft Teams) held in the first half of 2022 and transcribed verbatim. The meetings were moderated by a research assistant (SIJ) with support from 2 psychology students. The focus groups were conducted in Swedish, professionally translated into English, and subsequently analyzed in English by nonnative speakers. The focus groups followed a semistructured format (see Multimedia Appendix 3 for the full interview guide) with 2 distinct objectives: to identify *general health needs* and generate user input on *the digital treatment content and design*. This study focused on the user-centered input on the digital treatment, and user input on general health needs will be presented in another publication. To facilitate concrete feedback, participants were given access to the treatment before the focus groups and asked to focus on the first 2 modules of the treatment and the general treatment vision.

#### Data Analysis: Focus Groups

Sample characteristics were analyzed using descriptive statistics. A qualitative framework analysis [46] was used for the (qualitative) focus group data using the following steps: (1) *familiarization* with the data by listening to recordings and reading transcripts, (2) *identification* of the thematic framework by deciding whether to conduct an inductive or deductive approach, (3) *indexing* by creating a coding frame of the highlights from the data, (4) *charting* by placing themes into rows and columns in summary, and (5) *mapping and interpretation* by looking into similarities and dissimilarities between participants’ responses.

The Consolidated Framework for Implementation Research (CFIR) [47] was used to guide the coding of the focus group data. The CFIR consists of 5 main domains (ie, intervention characteristics, outer setting, inner setting, individual characteristics, and implementation process) [47,48]. Phase 1 focused on the CFIR domain *intervention characteristics*, specifically on the design and content of the digital behavioral intervention, including the subdomains of evidence base, relative advantage, adaptability, complexity, and design of the innovation (which refers to the “digital intervention”; see specific definitions in Multimedia Appendix 4). In total, 2 independent researchers (AT and SLB) conducted the qualitative analysis, with a third researcher validating the outcome of the analysis by reviewing the final framework.

### Phase 2 (Testing): Piloting the Digital Intervention

On the basis of feedback from the focus group participants in phase 1, the prototype was refined and prepared for testing in phase 2 to be carried out through piloting the digital intervention with end users. The objective of this phase was to test and identify areas for improvement of the digital treatment design (ie, content, structure, and format).

#### Participants and Recruitment

The inclusion and exclusion criteria for patients and therapists in phase 2 were consistent with the eligibility criteria of the focus groups (phase 1; see the Patient and Recruitment section). In addition, patients were excluded if they (1) had an injury or illness that required an immediate assessment or treatment, (2) had changes in prescribed medication in the previous 3 months or changes were expected in the following 3 months, or (3) had received CBT treatment during the previous 6 months. Participants (n=11 patients and n=3 therapists) were recruited from the same 2 regions (Stockholm and Kalmar).

#### Materials and Procedures

##### Overview

The DAHLIA treatment (prototype version 1.0) consisted of 4 self-guided microsessions per week for a total of 24 sessions delivered over 6 weeks. Moreover, patients had weekly contact with a therapist through a 30-minute phone or video call. The digital intervention was offered through the Swedish health care system implemented into a digital system called 1177, the national health care web platform in Sweden [49], in collaboration with health care providers from Region Stockholm and Kalmar and health care developers and digital designers in Region Kalmar and supported by the industry partner Inera for maintenance. The 1177 platform is a secure system that ensures confidentiality via Sweden’s public e-identification systems (BankID, Freja eID, and Foreign eID).

The intervention was delivered via 1177’s *Stöd och Behandling* (support and treatment) feature, which is designed to support digital care programs. While the platform provides a structured framework enabling health care providers to deliver digital care programs, the technical architecture imposes certain constraints, especially on the design and customization. These constraints include limited flexibility in the user interface design and multimedia services. Therefore, interactive features (eg, videos and exercises) could only be implemented through preexisting templates or external resources. In addition, as the platform does not support real-time communication (ie, video calls and phone calls), each region complemented the intervention by using its secure internal systems to conduct these interactions.

##### Evaluation of Engagement With Treatment

Every week, at the end of the contact with their therapists, patients were asked to evaluate the module based on how helpful, enjoyable, and comprehensible they perceived it to be by responding to a set of statements (eg, “I experienced this week’s session as helpful/enjoyable/understandable”) rated on a 7-point scale ranging from 0 (not at all) to 7 (very much). Furthermore, after completing the intervention, a semistructured exit interview (see Multimedia Appendix 5 for the full patient exit interview guide and Multimedia Appendix 6 for the full therapist exit interview guide) was conducted with each participant (ie, patients and therapists) by a researcher (LE, SP, SLB, and AST) to assess user experiences of engaging with prototype version 1.0, focusing on overall experience of the treatment (eg, Did the digital treatment interfere with your daily routines? Would you recommend this treatment to a friend with a similar condition?) and more specific questions on microsessions and weekly contact with their therapist.

In addition, therapists were interviewed about their experience of providing the intervention (eg, Was it easy to navigate the digital treatment? Was the frequency of communication with the patient adequate?). These evaluations and reflections from end users, including treatment patients and therapists, were intended to guide the researchers in revising the treatment’s design (structure, content, and format). Overall, these evaluations were chosen to explore intervention and implementation success (see the Materials and Procedures section). The exit interviews were held in either Swedish or English, professionally translated into English when conducted in Swedish, and subsequently analyzed in English by nonnative speakers.

#### Data Analysis: Pilot-Testing

All quantitative data were analyzed using descriptive statistics (mean, SD, and range). The same stepwise approach as in phase 1 (Intervention Structure for Prototype Version 0.0 section) was used to analyze the qualitative data [46,47]. Specifically, the *implementation process* domain of the CFIR [47] was used as codes to analyze the qualitative data from patients’ and therapists’ weekly evaluations and exit interviews. For this analysis, the CFIR *reflecting and evaluating* construct within the *implementation process* domain was used to explore the end users’ perceived intervention and initial implementation success (see specific definitions in Multimedia Appendix 7).

## Results

The results of each phase (0=preparation, 1=design, and 2=testing) are presented in the following sections.

### Phase 0 (Preparation): Relevant Patient Characteristics, Needs, and Treatment Targets for the Digital Intervention to Guide the Preparation of the Prototype (Version 0.0)

**Figure 2:**
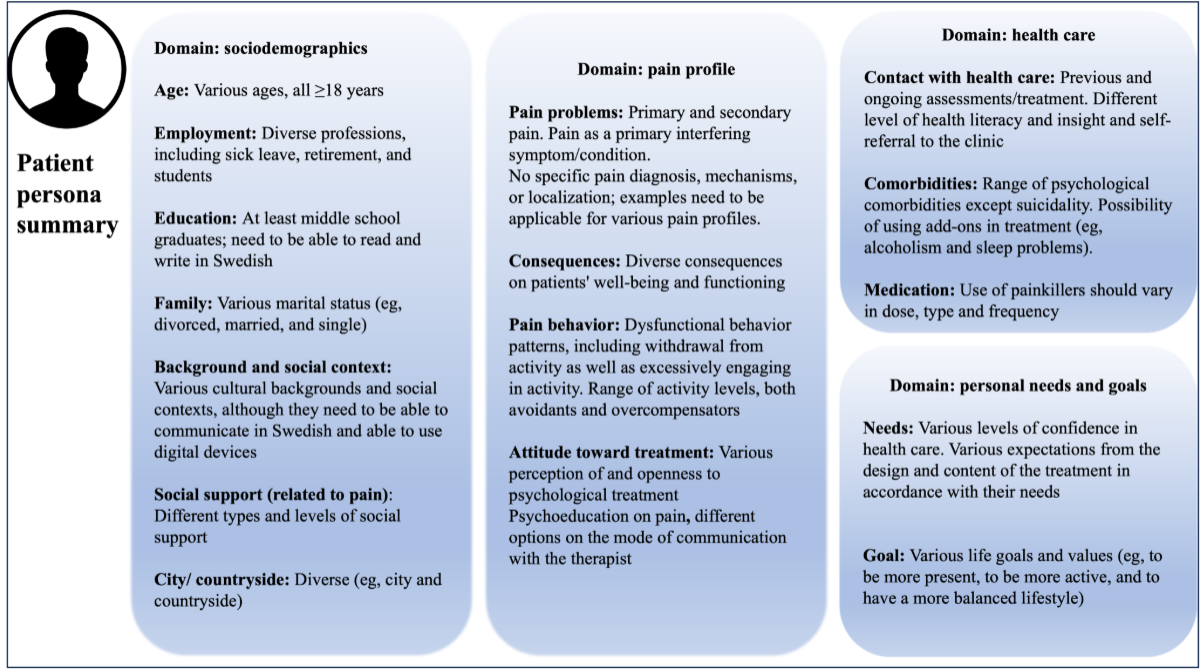
Summary of implications of patient personas on the development of the DAHLIA digital behavioral intervention for people with chronic pain.

Considering the literature and clinical experiences, the group of researchers and clinicians found it helpful and sufficient to use 3 patient personas to describe patient characteristics, needs, and treatment targets. Across all patient personas, the four domains included (1) sociodemographics, such as educational level, work, family, background, social environment, and living location; (2) pain profile, including pain problems, impact of pain, pain behavior, and attitude toward the treatment; (3) health care, such as contact with health care, comorbid conditions, and medications; and (4) personal needs and goals, particularly those related to treatment. The synthesis of characteristics, needs, and treatment targets across all 3 patient personas resulted in a visual summary (Figure 2). Moreover, based on the patient personas, the group identified a number of key challenges and ways to approach these challenges in the design and testing phases, which are summarized in Table 1.

**Table 1:** Key challenges identified by using patient personas for the development of the DAHLIA digital behavioral intervention for people with chronic pain and potential ways to approach these challenges.

Key challenge	Approach
The chronic pain population is heterogeneous due to various characteristics, including age, gender, pain history, needs, comorbidities, abilities, and ethnicity.	The content needs to be written using inclusive language (eg, easy reading level) and feature diverse examples, the treatment needs to be tested in a heterogeneous sample, and subgroup analysis might be necessary to explore which specific characteristics influence treatment outcomes.
Digital literacy will vary significantly as some patients will be reluctant or not able to use technology.	A nondigital alternative and technology training in the onboarding phase (during enrollment) need to be offered.
The varying needs and expectations in this heterogenous population may require flexibility regarding treatment content.	The theoretical foundation of the treatment (see the Theoretical frameworks and conceptual model section) is transdiagnostic and a standardized prototype treatment and, therefore, can be assumed to benefit the population, but tailoring or add-on elements may be needed to provide support for patients with specific or more complex health challenges.

### Phase 1 (Design): Preferences of End Users for the Design (ie, Content, Structure, and Format) of the Digital Intervention

#### Characteristics of the Participants

##### Patient Focus Groups

A total of 7 patients provided informed consent, resulting in 4 (57%) patients in the first focus group and 3 (43%) patients in the second focus group. Despite rescheduling both groups once, 1 patient per group was unable to join (reasons for dropout: sickness and physician’s appointment). Consequently, 71% (5/7) of the patients participated in the focus groups (3/5, 60% in the first focus group and 2/5, 40% in the second focus group). Participants were, on average, aged 43.6 (SD 7.8; range 37-53) years, 20% (1/5) identified as men, and 80% (4/5) identified as women. The educational levels of the participants ranged from high school diploma to college or university degree (3/5, 60% high school diploma; 1/5, 20% ongoing university studies; 1/5, 20% college or university degree). All participants (5/5, 100%) reported having chronic pain, 60% (3/5) were unsure or had not received a specific pain diagnosis, and 40% (2/5) reported being diagnosed with Ehlers-Danlos syndrome (of whom 1/2, 50% reported a fibromyalgia diagnosis as well). Using a numerical rating scale from 0 to 10 [2], participants reported a current average pain level of 5.8 (SD 1.5; range: 4-8) and an average pain level during the previous week of 6.6 (SD 1.14; range 5-8). A summary of the characteristics of the patients is provided in Table 2.

**Table 2:** Sociodemographic and pain-related characteristics of the patient participants involved in the focus groups during the design phase of the DAHLIA digital behavioral intervention for people with chronic pain (N=5).

	Patient 1	Patient 2	Patient 3	Patient 4	Patient 5	Patient 6	Patient 7
Participation	Yes	Yes	Yes	Yes	Yes	Dropout	Dropout
Age (y)	40	37	53	37	51	47	—^a^
Gender	Woman	Man	Woman	Woman	Woman	Woman	—
Educational level	High school diploma	High school diploma	Ongoing university studies	High school diploma	College or university degree	High school diploma	—
Pain diagnosis	Ehlers-Danlos syndrome	Unsure or did not know	Undiagnosed pain	Ehlers-Danlos syndrome and fibromyalgia	Undiagnosed	Ehlers-Danlos syndrome	—
Current pain level (from 0 to 10)	6	4	5	6	8	6	—
Pain level during the previous week (from 0 to 10)	7	6	5	7	8	7	—

^a^No data available for patient 7.

##### Therapist Focus Groups

A total of 3 focus groups with 4 therapists each were conducted. In total, 12 therapists (n=8, 67% from Region Kalmar and n=4, 33% from Region Stockholm) provided informed consent and joined the focus groups (12/12, 100% retention rate). The therapists were, on average, aged 42.8 (SD 12.0; range 29-64) years, 25% (3/12) identified as men, and 75% (9/12) identified as women. All participants were licensed psychologists with, on average, 12.3 (SD 9.4; range 2-31) years of clinical experience and 9.5 (SD 9.8; range 0-28) years of experience working with patients with chronic pain.

#### Outcomes of the Patient and Therapist Focus Groups

The focus groups provided input on the treatment relevant to the CFIR *intervention* domain and its related constructs (evidence base, relative advantage, adaptability, complexity, and the design; see the specific definitions in Multimedia Appendix 4), which is presented in brief in the following sections.

##### Evidence Base of the Treatment

Therapists expressed that they perceived the content as relevant, valid, and evidence based. Patients did not have any input regarding the evidence-based content.

##### Relative Advantage of the Treatment

Patients and therapists reported different advantages and disadvantages in relation to other types or formats of treatment, which are presented in Table 3 in detail and summarized in this section. Some patients reported that group-based treatment had an advantage over individual treatment as it facilitated the exchange of experiences with others, whereas others endorsed individual treatment as the pace could be adjusted to the patients’ needs. It was also mentioned that digital delivery may be challenging for older adults who struggle with technological devices. Furthermore, therapists reported that the digital self-help format with preexisting content and structure enabled the delivery to be more concise than standard treatment, that the microsession format was preferable over longer modules, and that the digital treatment format could be introduced to the patients earlier and cover a broader geographic area (ie, rural areas) than traditional approaches.

**Table 3:** Perceived relative advantages of the digital behavioral intervention for people with chronic pain as identified by patients (n=5) and therapists (n=12) in qualitative focus groups.

Participant group and subcategory—relative advantage		Example quotes
**Patients**		
	Pros and cons of individual vs group-based treatment	“I think that’s a good thing: if someone has difficulties with something that might not be so easy to deal with in a group, then there might be an opportunity for private, individual sessions that.” [Patient 2] “It’s always good to exchange experiences with others I think.” [Patient 5]
	Potential disadvantage—digital delivery (eg, older age and low socioeconomic status)	“[Father of participant] was born in 50s’, hates technology and everything surrounding it, doesn’t even have a computer and refuses to get one. Is there any kind of technology help? What is required to do these programmes? Do you need a computer or is it enough to have a phone? How do you deal with those on welfare, if they can’t afford a smartphone and computer?” [Patient 4]
	ACT^a^ preferred over traditional CBT^b^	“I’ve tried CBT. I’m not a big fan of CBT, to be honest. I put more stock in ACT. Acceptance, which is something I’ve worked really hard on because I’ve been a bit black or white.” [Patient 5]
**Therapists**		
	Digital treatment enables therapists to provide knowledge to the patient in a more structured way than face-to-face or telephone-based treatment	“I personally would have had a more difficult time if [I] met the patients face-to-face in this type of treatment without any support... Having a treatment program that you follow, I feel that the knowledge I have [then] about chronic pain is good enough.” [Therapist 1] “This kind of program [is not so bad], because there is built-in structure and the knowledge that the patient is getting, is what the patient needs so that it doesn’t depend as much on my structure.” [Therapist 4]
	Digital treatment offers more training options to therapists (than currently available)	“It is difficult to become good at something that requires a lot from the practitioner.... I think that a program like this can fulfil such a [training] function.” [Therapist 4]
	iCBT^c^ for chronic pain currently not available	“It’s worth developing this because it’s needed.” [Therapist 2]
	Microsession format potentially advantageous over longer iCBT sessions	“It’s very different from the regular internet-based CBT we [therapists] work with but [when] you get the patient to understand to go in ten minutes every day, then it becomes more of a habit.” [Therapist 7]
	Digital treatment could be offered earlier to patients than traditional therapy	“Internet-based [treatment] is one of the first steps.... [If the system would] be able to [refer patients at] an earlier stage and in that case, we would be able to be helpful with patients that we don’t see today.” [Therapist 7]
	Digital treatment could cover a wider area than traditional therapy	“The possibility of practicing because I have the whole region as a ‘catchment area.’ It’s really good to be able to reach out in this way [digitally].” [Therapist 5]

^a^ACT: acceptance and commitment therapy.

^b^CBT: cognitive behavioral therapy.

^c^iCBT: internet-based cognitive behavioral therapy.

##### Adaptability of the Treatment

In both patient and therapist focus groups, participants discussed the mode of communication for the weekly therapist-patient contact. While therapists emphasized the benefits of an asynchronous messaging function over a live chat function and expressed potential preference for phone calls over video calls, patients expressed a general preference toward video calls over phone calls or messages. The varying views and preferences imply a need for flexibility in the mode of communication.

Furthermore, therapists suggested that the digital treatment should be adaptable according to patients’ level of knowledge and interest as some patients might have sufficient pain education whereas others would benefit from additional information. The therapists argued that the treatment length might also need to be adapted to symptom complexity; for instance, patients with longer and more complex pain and health issues might benefit from longer treatment periods, and the frequency of contact should be flexible, as highlighted by therapists. Finally, both patients and therapists proposed a text-to-speech function and subtitles in videos to better accommodate people with disabilities such as hearing, visual, or cognitive limitations. Details and supporting quotations can be found in Table 4.

**Table 4:** Adaptability aspects of the DAHLIA digital behavioral intervention for people with chronic pain as identified by patients (n=5) and therapists (n=12) in qualitative focus groups.

Participant group and subcategory—adaptability			Example quotes
**Patients**			
	Flexibility in mode of communication; preference for video calls	“There’s someone who sees me. Once a week, I know I’ll be seen.” [Patient 3] “I think you should be able to choose.... You might feel that’s enough writing for today. I don’t have anything to say but today I want to talk on the phone because I don’t feel I can sit at the computer [and type a message]. So I can make the call while sitting in the car, taking the bus. But then I might feel like, now I want to see another human being [and I chose a video call].” [Patient 4]	
	Flexibility in engagement with treatment (ie, choosing certain exercises)	“That maybe there’s the possibility to adapt, if there’s somebody who’s having a very hard time, [then] you don’t have to do all of this, but we can just do a specific part. To be able to adapt it to the individual a little bit because in my experience [of those who] have chronic pain, we are all different with different functions, different personalities, basic stages, other difficulties. I think it would do some good to be a bit flexible from person to person.” [Patient 2]	
	Text-to-speech function and subtitles in videos	“Is it possible to have the text read aloud, or will this be made possible?... text-to-speech and audio description or subtitling are a must I think.” [Patient 4]	
	Inclusivity toward people with disabilities	“Because if you do as much preparatory work as possible, e.g., you put in a glossary, you prepare for audio description and subtitling and text-to-speech and everything, because if you have a good foundation, the target group doesn’t really matter because then you can just update.” [Patient 4]	
	Mobile and computer versions	“You can make one [version of the program] that’s a mobile version but with even less text and even more in summary form, but still the same content.” [Patient 4]	
	Provision of technical support if needed	“But will they be any kind of technical help then [for those who need it]?” [Patient 4]	
	Paper-based version (if technology is not usable and) as a long-term resource	“You [could] have some kind of physical book available with information and everything that you can go back to and read parts of.... You’ve undergone the treatment, you received the information, but five-ten years later the time may be ripe to put it to use and then you still have the book.” [Patient 4]	
**Therapists**			
	Digital treatment needs to be adaptable to patients’ abilities and disabilities (eg, linguistic and cognitive) and preferences	“Pain patients often have difficulty with concentration and with reading a text. If an audio file could be added for each text, so that [the patient] can press [play] on it [and] there is a voice that says exactly the same things as in the text so [the patient] can both listen and somehow follow the text.” [Therapist 10]	
	Digital treatment should be adaptable according to patients’ level of knowledge and interest	“If you [patient] want to delve deeper, then, maybe have something that you can activate and read on if you want more information about it... Maybe some patients already know what pain is and want to go directly do the exercises... make it more efficient.” [Therapist 3]	
	Treatment length might need to be adapted to symptom complexity	“I think that pain is very complex, and it can take time to implement behavioral changes... if you want to implement a proper behavioral change to something that has been going on for thirty years, six weeks may be a very short time.... It depends on which patient group you think should be included in this [treatment]. Is it really severe pain patients or is it primary care patients with a little milder pain who haven’t had pain for so long? Then I think that then it [the 6-week duration] is quite reasonable.” [Therapist 5]	
	No space limitations on patients’ response to exercises (ie, word limit)	“[The patient could] have the possibility of filling in more.” [Therapist 10]	
	Flexibility in frequency of contact	“With some patients, you maybe have many phone calls while with others you may have nothing in the meantime but a follow-up at the end or something in the middle and then at the end. It can be varied and timewise it is also a bit different.” [Therapist 7]	
	Flexibility in mode of communication (ie, telephone, video, and messaging)	“Let them choose when we book an appointment: should I call you up or do you want to write in the chat function if you want some feedback on any particular part of it [the exercise].” [Therapist 2]	
	Reason to provide flexibility in mode of communication—scheduling (patients and therapists)	“It’s better [for one’s own structure] to have a clear that this is the feedback day. This is when you can expect to get a response from me.... They know that there is a specific day and that we have a contract at the beginning that this is what the agreement looks like.” [Therapist 4]	
	Reason to provide flexibility in mode of communication—time for preparation (therapists)	“You have to be very aware of what [the patients] have worked on because it is difficult to go [into the treatment] and have video [the camera on] at the same [while talking online face-to-face with the patient].” [Therapist 8]	
	Reason to provide flexibility in mode of communication—therapists encourage use of the messaging function (therapists)	“We always encourage people doing internet-based CBT to write to us in this messaging function.... There are quite a few who never write in that box no matter how much you encourage them. [Therapists could have personal contact regularly].” [Therapist 7]	
	Reason to provide flexibility in mode of communication—preference for phone calls (patients) and video not being superior to phone calls (therapists)	“If you choose only chat function then it is very difficult... Video is also an option, if it works. You often waste a lot of time if it’s not working, and then phone always works.” [Therapist 3]	
	Asynchronous messaging (not live chat) function	“I write answers and feedback based on what they [patients] wrote previously, not that it is a live chat.” [Therapist 8]	

##### Complexity of the Treatment

Concerning the complexity of the treatment, patient focus groups suggested that the amount of text in the digital treatment could be challenging for some individuals and should be reduced to improve feasibility and user-friendliness. In addition, bullet points and summaries could enhance clarity, and exercise instructions should be presented clearly for better understanding.

Therapists further reported that the treatment could be considered demanding for both patients and therapists.

##### Design of the Treatment

Within the CFIR construct “design,” 2 subthemes were identified: “design elements,” which refers to technology, branding, media, text, and aesthetics, and “content elements,” which refers to reflections from participants on how the content or treatment elements delivered should be improved or what should be added or removed.

###### Design Elements of the Treatment

**Figure 3:**
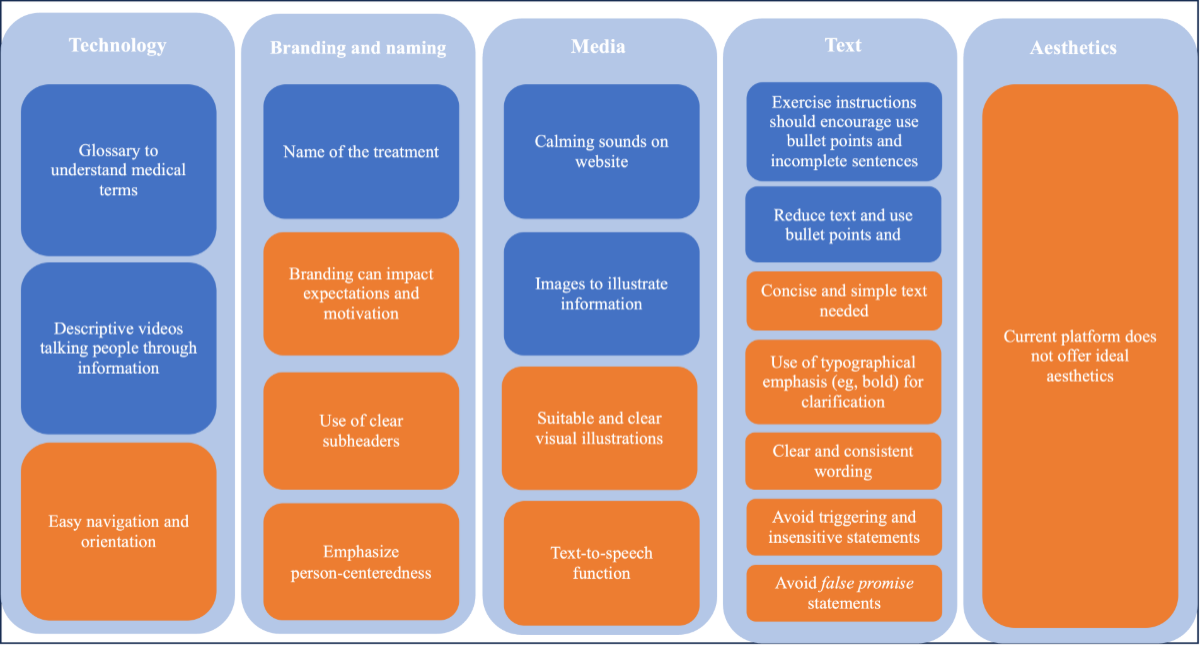
Summary of preferences and suggestions for the design elements of the DAHLIA digital behavioral intervention for people with chronic pain as identified by the focus group participants (blue boxes=patients [n=5]; orange boxes=therapists [n=12]).

Figure 3 summarizes preferences and suggestions on the design of the digital intervention from patients and therapists.

###### Content Elements of the Treatment

Table 5 presents preferences and suggestions from participants regarding the content of the digital intervention, which is also summarized in this section. The treatment program (prototype 0.0, with suggestions for the full version) was evaluated as well structured by therapists. Therapists suggested having a table of contents and summaries at the beginning of the treatment, including psychoeducation on pain, and inclusive and diverse examples. Moreover, therapists highlighted the importance of the content fitting diverse patient groups, potentially targeting behaviors such as pacing or overcompensating.

Patients suggested that the content, including exercises, should ideally be tailored to individuals’ needs, such as involving sleep-related content for people who struggle with insomnia and assessing treatment expectations. Additional material to the treatment content could potentially be offered through external links and further readings. Patients suggested 1 or 2 sessions as a “booster” after the completion of the intervention period as a reminder and relapse prevention. Patients also mentioned the importance of content that focuses on enabling a meaningful life, for instance, focused on how to find things that bring feelings of happiness and contentment in life by identifying things that are important for people and targeting (mourning of) the loss of function and realization of the current capacity. Further details and example quotes are presented in Table 5.

**Table 5:** Preferences and suggestions from focus group participants (n=5 patients and n=12 therapists) regarding the structure and content elements of the DAHLIA digital behavioral intervention for people with chronic pain.

Focus group and subcategory—design (specifically the content)		Example quotes
**Patients**		
	Sleep-related content	“I’m thinking more about how to sleep, what you can do in bed, how to relieve things in the best way to get a better sleep.” [Participant 3]
	Several booster sessions	“I would recommend two [booster sessions].” [Participant 3]
	Asking patients about treatment expectations	“[Talking about expectations] makes it easier to move on to the next step, because I’m open with what I expect and where I come from, what I’ve chosen not to do and how it’s affected me.... And if you are honest and say that this is what I expect, then the person who meets me can be honest and say this is what we can provide you with.” [Participant 1]
	Focus on enabling a meaningful life	“That’s what I’ve been missing. Getting back a little more, meaning to live life instead of surviving it. To be able to find fun things to feel content, happy and satisfied.” [Participant 2]
	Focus on life values	“I think it’s important to reflect and look at what works for me in everyday life, and what it is that drives me in life. You may sometimes think it’s really hard to have a family when dealing with pain, but at the same time, they have been a driving force. They are my motor. I also have my work, which I really enjoy, and I have an understanding employer and all my co-workers know that I am sometimes in pain. And I think it’s important to take the time to stop and reflect [though these exercises] and realize, today is a bad day, but I can still make things work.” [Participant 5]
	Focus on (mourning) loss of function and realizing current capacity	“You mourn yourself; you’ve passed away in some way.... You had a hard time finding your purpose again.” [Participant 1]
	Microsessions likely to be suitable	“[Microsessions are] just enough. More than that would be too much. It then becomes like homework.... you can squeeze it in here and there... 10 minutes is easier to fit into life.” [Participant 1]
	Optional extra material	“[The program is] good as it is but you might want the opportunity to be able to understand a bit more. You [could] link to a page where you can read more about [a certain topic]. There should be some opportunity to delve deeper.” [Participant 4]
	Body scans and meditative exercises	“For each day, you might have the option of doing one of those body scans.... For those panic attacks where my body stops working. I couldn’t walk for six months and [doing a body scan] was the only thing that got me to learn to walk again. I became aware of my body.” [Participant 4]
	Reuse of existing content sources	“I’ve learned when doing these projects that it’s unnecessary to make material that already exists.” [Participant 4]
**Therapists**		
	Structured program	“It feels really good to finally have a structural treatment program.” [Therapist 1]
	Emphasis on exercise and skill training	“[Therapists] train the [patients] to observe, and that is a skill to learn, [to] observe nuances as well. Not everything is [about] pain, it might also be other things, [e.g., tiredness].” [Therapist 10]
	Mandatory reflections on exercises	“[Currently,] you can write to your practitioner how you perceived [the exercise] and I think that is valuable information... maybe not leave it optional.” [Therapist 1]
	Pros and cons of stating duration and word count per exercise	“A detail that bothered me a lot, is that it says how long the exercises take, it takes 2 minutes to complete this one [exercise]. I don’t understand the purpose of that. I think that each patient will be very individual.... It can still be good to have a short [text] ‘here do a short reflection’... should write two sentences or two hundred.” [Therapist 1] “Super great thing that it says four minutes [to do this session] and [the patient might think] yes I have that [time right now], so I think that is good.” [Therapist 10]
	Microsessions	“It’s good that [the treatment] seems to be very brief.... This whole idea of taking small sections every day instead of longer pieces [like in other iCBT programs] might be a good adaptation for these patients who usually have much lower energy.” [Therapist 5]
	Importance of first exercise	“It’s good that [the treatment] takes values and goals into account early.” [Therapist 5]
	Table of contents and summaries	“It would have been nice to have a bit more of a summary that sums up all these sections.... For the patient to keep track of all these things.” [Therapist 5]
	Fittin diverse patient groups (ie, pacing vs overcompensating)	“The material seems to be about a patient [with more] fear of movement, but many of these patients are over compensators who are angry and struggling with their pain thinking ‘I won’t let the pain win’ and they do too much.” [Therapist 6]
	Inclusive and diverse examples as part of exercises	“[The patient] can press on [a symbol] and then [they] get more information about an exercise [and] what [they] can answer in this exercise. I think it would be possible to have a few more examples and maybe a few more pain-related examples.... Pain patients can sometimes have difficulty with generalizing. If I [as the therapist give an example about] pain in my knee, then a patient who has back pain will say ‘but then this doesn’t apply to me.’ You often need to have a lot of variations of examples so that the [patients] will be able to recognize themselves.” [Therapist 10]
	Psychoeducation on pain	“[The program should] have an explanation [on the] physiology of chronic pain to understand why [is the patient] doing this [program]. Many [patients] can be quite preoccupied with [the idea of] what is wrong with [their] body and finding [a] cause behind it. [the program needs to] explain that there is a very big difference between acute and chronic pain, they are driven by completely different mechanisms.” [Therapist 9]
	Asking patients to report limitations at the start of treatment	“At the beginning of the treatment, the patient had to report ‘Do I have any limitations, or have I been told that there is something I shouldn’t or can’t do?’ [It is difficult as a therapist] having to play detective and find out and the patient can also have more concerns, but then you can discuss things.” [Therapist 6]
	Inclusion of more complex ACT^a^ processes (eg, self as context)	“Internet-based treatment is now mature enough to take a step further and include more [ACT] processes... maybe self as a context. It [this process] is really hard to grasp even as a therapist.” [Therapist 6]

^a^ACT: acceptance and commitment therapy.

##### Summary of Key User Feedback

To enhance clarity and facilitate interpretation, a summary table was created to illustrate how example key user feedback was mapped to relevant constructs within the CFIR *intervention characteristics* domain (Table 6).

**Table 6:** Summary of selected user feedback from focus groups (n=5 patients and n=12 therapists) mapped to Consolidated Framework for Implementation Research (CFIR) constructs—example insights for intervention design.

CFIR construct	User feedback	Explanation
Evidence base	Content was relevant and valid	Evidence-supported content was valued
Relative advantage	Microsession format was suitable	The microsession format was seen as an advantage in comparison to longer sessions
Adaptability	Flexibility in mode of communication	Weekly patient-therapist contact should be adaptable to different communication methods (eg, video or phone calls and messaging)
Complexity	Desire for reduced text and use of bullet points and summaries	Simpler and more concise text was suggested, using bullet points and summaries to improve clarity
Design	Request for visual aids and audio options (eg, images, videos, and text-to-speech function)	The intervention can be more engaging and accessible through multimedia elements

### Phase 2 (Testing): End Users’ Perceptions of Engaging With the Treatment Prototype and Areas for Improvement

#### Overview

On the basis of the focus group findings, the intervention prototype version 1.0 was created by 3 researchers with clinical expertise (RKW, LE, and IF). The treatment consisted of 6 modules, including weekly contact with a therapist where patients and therapists together chose the mode of communication (phone or video call) as well as 2 booster sessions (after 2 and 4 months). Each module included 4 microsessions, resulting in a total of 24 microsessions. An overview of 1 module and a screenshot of an exercise are provided in Multimedia Appendix 8.

Each new module was enabled by the therapist after the previous module was completed; while it was suggested that patients complete 1 module per week, flexibility was allowed. Suggestions from the focus groups that were not implemented in the treatment prototype version 1.0 are presented in the End-User Suggestions for Further Treatment Improvement section.

#### Participant Flow and Characteristics

**Figure 4:**
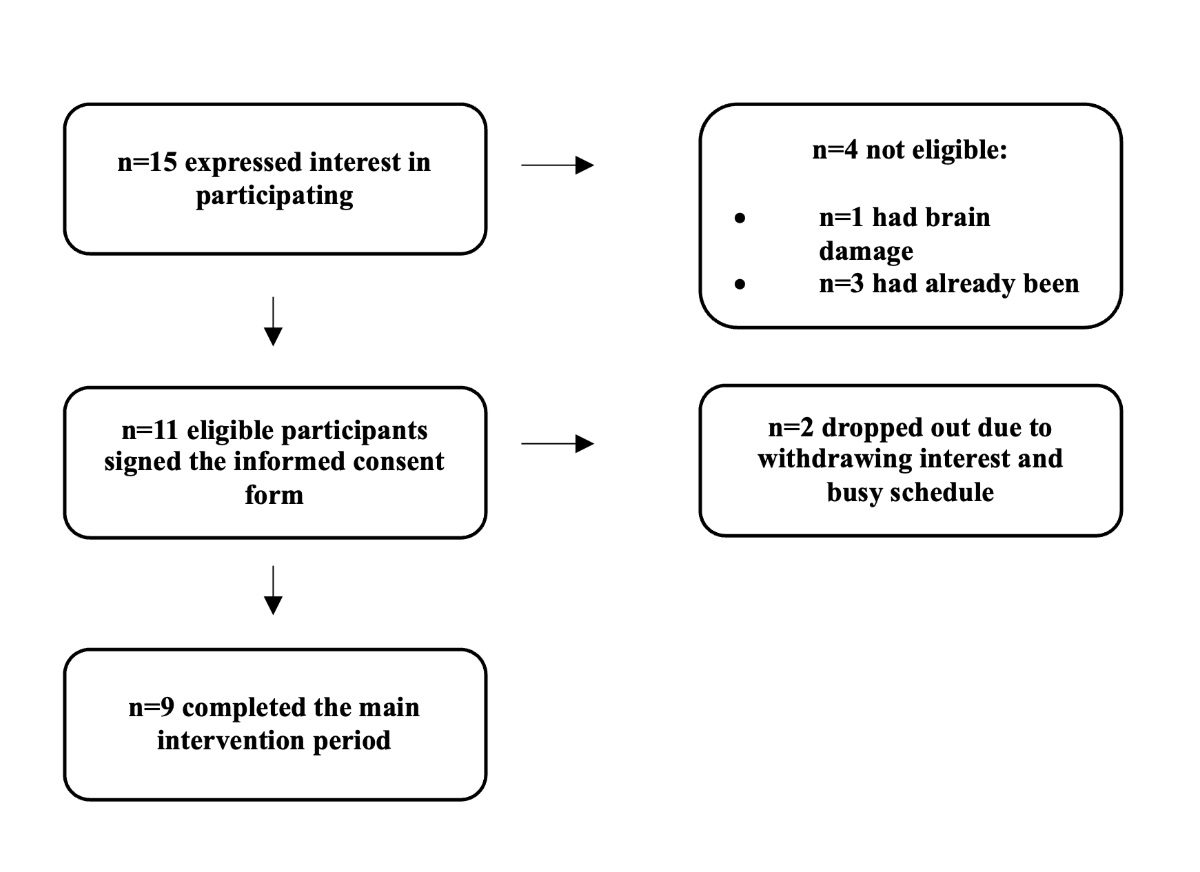
Patient flow in the piloting of the DAHLIA digital behavioral intervention for people with chronic pain (phase 2).

For phase 2 (testing), 15 patients expressed interest, of whom 4 (27%) were not eligible due to ongoing pain treatment or rehabilitation (n=3, 75%) or brain damage (n=1, 25%). Thus, 11 patients (n=8, 72% from Region Stockholm and n=3, 27% from Region Kalmar) provided informed consent and started the treatment. A total of 18% (2/11) of the participants dropped out, and 82% (9/11) of the patients completed all modules (see Figure 4 for details). Of the participants involved in the focus groups, 17% (2/12) of the therapists and no patients also took part in the pilot-testing, and the remaining pilot participants were newly recruited.

Patients were, on average, aged 46.9 (SD 6.9; range 36-58) years, 18% (2/11) identified as men, and 82% (9/11) identified as women. Educational levels ranged from high school diploma (3/11, 27%) to college or university degree (2/11, 18% ongoing university studies; 6/11, 55% college or university degree). At baseline, numerical rating scale scores (0-10) showed that the current pain level was, on average, 5.1 (SD 2.3; range 3-10) and the mean pain level in the previous week was 5.6 (SD 2; range 3-10). Patients had various diagnoses—migraine, chronic fatigue syndrome, herniated disc, arthritis, fibromyalgia, complex regional pain syndrome, and hypermobile Ehlers-Danlos syndrome—and 9% (1/11) of the participants did not know their pain diagnosis.

In total, 3 licensed psychologists delivered the treatment (n=2, 67% from Region Stockholm and n=1, 33% from Region Kalmar). The therapists were all women (3/3, 100%) and had a mean age of 47.6 (SD 11.06; range 36-58) years, with an average of 21.7 (SD 13.31; range 7-33) years of experience providing psychological therapy, 18.3 (SD 14.6; range 2-30) years of experience working specifically with people with chronic pain, and 12 (SD 9.84; range 1-20) years of experience using acceptance and commitment therapy.

#### End-User Perceived Success of the Intervention

The success of the intervention refers to the direct reflection and evaluation of engaging with the intervention. As such, patients rated the modules as very helpful (mean 5.1, SD 1.69; range 1-7), moderately enjoyable (mean 4.3, SD 1.66; range 1-7), and very understandable (mean 5.4, SD 1.54; range 1-7). In addition, qualitative feedback was provided. Input varied, resulting in different suggestions for improvement (Table 7).

**Table 7:** Patient evaluations of the weekly modules during pilot-testing (phase 2) of the DAHLIA digital behavioral intervention for people with chronic pain, including ratings for helpfulness, enjoyability, and understandability, and selected example elaborations^a^.

	Helpful		Enjoyable		Understandable	
	Rating, mean (SD; range)	Example quote	Rating, mean (SD; range)	Example quote	Rating, mean (SD; range)	Example quote
Module 1 (n=11)	4.27 (2.05; 1-7)	“...a good start to understand pain, however, a bit more theoretical”	4.81 (2.08; 1-7)	“...fun and motivating to continue”	5.45 (1.80; 2-7)	“...easy to understand”
Module 2 (n=9)	4.89 (2.20; 1-7)	“...already thought about the things”	4.56 (1.94; 1-7)	“...it is good that the module is difficult”	6 (1.30; 3-7)	“...instructions for the exercises were difficult to understand”
Module 3 (n=9)	5.27 (1.19; 3-7)	“...exercises led to a conversation with the therapist which in turn led to insights”	3.81 (1.53; 1-5)	“...the navigation in between exercises messy”	4.45 (1.86; 1-7)	“...highlights should be used for different exercises to make them more distinct”
Module 4 (n=9)	5.45 (2.01; 1-7)	“...difficulty with changing things in combination with the high load in life”	4.09 (1.75; 1-7)	“...satisfying to get new insights”	5.63 (1.36; 3-7)	“...more theoretical rather than practical”
Module 5 (n=8)	5.36 (1.20; 4-7)	“...a bit less concrete than earlier chapters”	4.63 (1.12; 3-7)	—^b^	5.45 (1.50; 2-7)	“...more difficult than the previous modules”
Module 6 (n=7)	5.36 (1.50; 2-7)	“...difficult and overwhelming due to the number of boxes that needed to be filled in each exercise”	3.90 (1.57; 2-6)	“...very difficult to navigate in between exercises”	5.54 (1.43; 2-7)	“...the exercises were massive”
Total	5.1 (1.69; 1-7)	—	4.3 (1.66; 1-7)	—	5.4 (1.54; 1-7)	—

^a^Perceived helpfulness, enjoyability, and understandability were scored on a scale from 1 (not at all) to 7 (very much).

^b^Not available.

Reflecting on the treatment as a whole during the exit interviews, patients considered the written material understandable and the text easily readable in terms of font size and formatting. A total of 4 sessions per week over 6 weeks were considered adequate, and the time needed to complete the sessions was acceptable.

Therapists evaluated the intervention as very beneficial for patients; they were overall satisfied and self-reported that the intervention was delivered as intended. All therapists expressed interest in delivering the intervention again in the future. The overall time investment to deliver the intervention was considered acceptable, whereas the time saved by delivering this treatment online was perceived as moderate. Details of patients’ and therapists’ perceived intervention success are provided in Table 8.

**Table 8:** Exit interview questions for patients (n=9) and therapists (n=3) on “reflecting and evaluating” the DAHLIA digital behavioral intervention for people with chronic pain following pilot-testing^a^.

Question			Rating, mean (SD; range)		Example quotes
**Patient feedback (n=9)**					
	“Did you experience the online treatment as helpful overall?”	5.75 (2.05; 1-7)		“I really think so. Mainly thanks to the sessions with my therapist. They were invaluable. Without them I am unsure of the value of the program.” [Patient D]	
	“Did you experience the online treatment as meaningful overall?”	5.88 (1.72; 2-7)		—^b^	
	“Was the written material understandable?”	5.50 (1.30; 3-7)		“First chapters were a bit tricky to understand, talked to my therapist and it got better.” [Patient F]	
	“Could you easily read the text in the treatment (ie, in terms of font size and formatting)?”	5.88 (1.80; 3-7)		“Font size and formatting were OK, but there could be bold and italic text formatting or different colors can be used to link parts together better. And [there should be] clickable links instead of having to go back and forth, that was messy! Extra difficult to do in the phone. Text-to-speech function would be an improvement.” [Patient A]	
	“Was the number of sessions per week (4 sessions) adequate?”	5.50 (1.41; 4-7)		“A good number of sessions per week, and you could do them more or less thoroughly, you could adjust to what is needed for you, personally.” [Patient B]	
	“Was the total number of sessions adequate?”	6.38 (0.91; 5-7)		“In general, six weeks felt adequate. During this specific period, it was a bit stressful. Don’t think that it is possible to do it in a shorter amount of time. But think that 6-8 weeks is optimal.” [Patient D]	
	“Was the time needed to complete the sessions acceptable?”	5.83 (1.16; 4-7)		“Sometimes it took a very long time, depending on how much writing I have done, or how much pain I have.” [Patient C]	
	“Did microsessions influence your behavior in everyday life?”	4.88 (2.58; 1-7)		“[The treatment] made me think about how I do things and how I can improve.” [Patient F]	
	“Did microsessions influence your emotions?”	4.75 (1.90; 2-7)		“[The treatment] made me think about things, in the back of my head.” [Patient D]	
	“Did microsessions influence your thoughts?”	5.38 (1.30; 3-7)		“I become more aware, was able to see myself from a different perspective.” [Patient C]	
**Therapist feedback (n=3)**					
	“Was the online treatment overall beneficial for your patients?”	6.33 (0.58; 6-7)		“For the majority of them it was very beneficial in different ways.” [Therapist B]	
	“How satisfied are you with the intervention overall?”	5.67 (0.58; 5-6)		“I like that it is 6 weeks, quite easy to understand. Seems to be inspiring for the patient. The therapist has to talk to patient every week and has to be prepared for the questions.” [Therapist A]	
	“Was the overall time investment to deliver the online treatment acceptable?”	5.67 (1.15; 5-7)		“It was well, and 6 weeks treatment is short, but also enough.” [Therapist A]	
	“Did delivering this treatment online save you time?”	3.67 (2.08; 2-6)		“Documentation and contact with participants take time similar to that of which I had seen them in person. However, [there is] greater flexibility in the digital format.” [Therapist B]	
	“Was the online treatment delivered as intended?”	7 (0; 7-7)		“I was lucky with my participants, they did what they were expected. It may not always be the case, everyone got the treatment in the intended time.” [Therapist A]	
	“Would you deliver the intervention again in the future?”	7 (0), 7-7		—	
	“What facilitated you to deliver the intervention?”	N/A^c^		“The protocol and the documentations helped a lot, contact with the supervisors helped a lot.” [Therapist B]	
	“What hindered you in delivering the intervention?”	N/A		“Nothing that I can think of, but it can be messy to set up time with the patients.” [Therapist C]	
	“What aspects of the intervention need improvements?”	N/A		“In this stage, module 3 should be clarified. All of my patients got stuck there, and did not understand. It seems like it is unclear that active pain patients have recommendations about being active, but they are already too active.” [Therapist A]	

^a^Responses were scored on a scale from 1 (not at all) to 7 (very much).

^b^Not available.

^c^N/A: not applicable.

#### End-User Perceived Implementation Success

The success of the implementation refers to the engagement with the wider treatment process (beyond the direct engagement with the treatment). As such, patients found the information regarding the use of the digital platform clear, experienced few technical problems, and considered the navigation of the digital platform easy. The weekly communication with the therapist was evaluated as very helpful, and the weekly meeting with the therapist was perceived as easy to schedule. Therapist contact was considered a very motivating experience, and patients mentioned that they felt supported by their therapist.

Similarly, therapists did not report any technical problems and found the platform very easy to navigate and the support for delivering the intervention, namely, training, technical guidance, and supervision, sufficient. The frequency of communication with patients and the time per interaction were very acceptable. Details on the end-user perceived implementation success are provided in Table 9.

**Table 9:** Exit interview questions for patients (n=9) and therapists (n=3) on “reflecting and evaluating” the implementation of the DAHLIA digital behavioral intervention for people with chronic pain following pilot-testing (phase 2)a.

Question			Rating, mean (SD; range)		Example quotes
**Patient feedback (n=9)**					
	“Did the online treatment interfere with your daily routines (work or other things)?”	3.88 (2.16; 1-7)		—^b^	
	“The treatment was delivered using a digital platform on 1177. Was the information about the digital 1177 platform clear?”	6.43 (0.53; 6-7)		“I was clearly guided during the briefing call before the intervention.” [Patient H]	
	“Was it easy to navigate the digital 1177 platform?”	5.38 (1.59; 3-7)		“Yes, but a bit difficult to find your way back to earlier exercises.” [Patient E]	
	“Did you experience any technical problems using 1177?”	0.88 (0.35; 0-1)		—	
	“Did you experience communicating with your health care professional as helpful overall?”	6.50 (0.92; 5-7)		“Absolutely, it was what I needed. Without it I do not know how much progress I would have made. Very meaningful!” [Patient D]	
	“Was it easy to schedule meetings with your health care professional?”	6.75 (0.70; 5-7)		“Very easy, my therapist was very helpful and flexible.” [Patient C]	
	“Did you experience communicating with your health care professional as motivating?”	6.50 (0.92; 5-7)		“Yes, my therapist has really made me think about things and given me new ideas.” [Patient F]	
	“Did you feel supported by your health care professional?”	6.50 (1.41; 3-7)		“Yes, in a way that I didn’t expect. I didn’t think that it would give so much and much of it was my therapist’s merit.” [Patient A]	
	“Would you consider the past 6 weeks ‘ordinary’?”	4.50 (1.77; 1-6)		—	
	“Did anything unusual occur during the treatment period?”	2.38 (2.26; 1-6)		—	
	“Would you recommend this online treatment to a friend with a similar condition?”	6.38 (1.18; 4-7)		“Already talked to a friend and recommended her.” [Patient F]	
**Therapist feedback (n=3)**					
	“Did you experience any technical problems using 1177?”	1 (0; 1-1)		—	
	“Was it easy to navigate 1177?”	7 (0; 7-7)		—	
	“Was the support for delivering the intervention (eg, training, technical guidance when issues arose, supervision) sufficient?”	7 (0; 7-7)		“I did not have any problems, but I felt very secure, it was not hard for me to receive help.” [Therapist C]	
	“Was the frequency of communication with the patient acceptable?”	7 (0; 7-7)		“It would be very time-consuming with so many patients, e.g. in primary care. It is not very frequent for written feedback but when you speak with them it is a little intense.” [Therapist A]	
	“Was the time per interaction (eg, phone call) acceptable?”	5.66 (1.15; 5-7)		“I thought 30 minutes [per interaction] would be tight, but on the contrary 30 minutes was perfect. Of course, some of them [the patients] exceeded but in general it was within 30 minutes.” [Therapist B]	
	“Did you feel prepared to deliver this treatment?”	6.33 (1.15; 5-7)		—	
	“Would you recommend the intervention to a colleague?”	5.67 (1.53; 4-6)		“I think it is a bit too early to say, but I am also drafting a couple of colleagues so we can say that I am already recommending; the intervention needs further elaboration.” [Therapist A]	

^a^Responses were scored on a scale from 1 (not at all) to 7 (very much).

^b^Not available.

#### End-User Suggestions for Further Treatment Improvements

**Figure 5:**
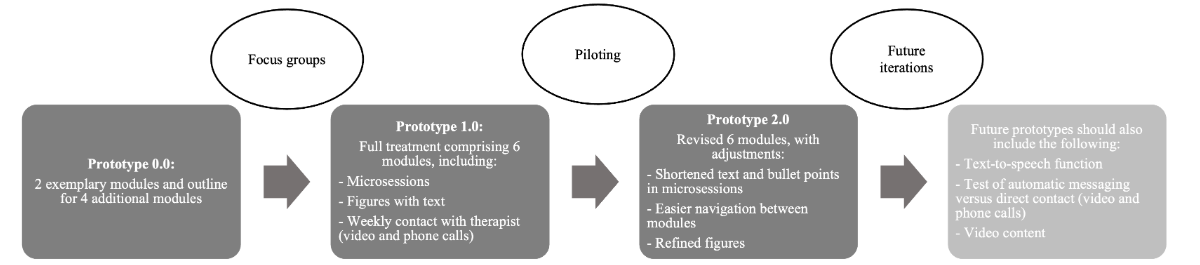
Integration of end-user feedback (patients and therapists) across prototypes in the development process of the DAHLIA digital behavioral intervention for people with chronic pain, highlighting how end-user feedback informed design adaptations over time.

When developing and improving the treatment, not all end-user suggestions could be integrated due to practical and technical reasons. The research team prioritized adaptations that could be addressed given the available time and financial resources. For instance, creating video materials is time-consuming and will benefit from collaboration with communication experts. Similarly, no automatic text-to-speech function is currently available in the platform, and alternatives need to be explored prospectively. Thus, taking all end-user input together, the following additional suggestions for improvements emerged in this study and require further refinement and testing to meet user needs: (1) creating video content and adding pictures or graphics to make the treatment more visually appealing; (2) integrating a text-to-speech function; (3) creating and integrating add-on materials, for instance, methods to improve sleep; and (4) facilitating navigation in between exercises. Moreover, according to participants’ views, it will be important to test whether the communication mode, namely, messaging only compared to phone or video calls, contributes to intervention and implementation success as user preferences differed. To illustrate how end-user feedback informed changes throughout the development of the DAHLIA intervention, Figure 5 provides a visual summary of feedback integration across prototypes.

## Discussion

### Principal Findings

This study aimed to clarify how a digital behavioral intervention can be developed through a user-centered approach to address the needs and preferences of the target population and the specific user input on the DAHLIA prototype 1.0 as part of the multiphase DAHLIA project. This paper presents data from the first iteration, which is considered part of the development phase. The following iterations are included in the evaluation phase, will be used to assess the feasibility and preliminary efficacy of the treatment, and will be presented elsewhere. The primary objective of this study was to develop a user-centered and evidence-based digital behavioral intervention for individuals with chronic pain by clarifying how the intervention should be designed in terms of structure, content, and format to address the needs and preferences of the target population.

This study involved 3 phases: preparation, design, and testing. The user-centered approach during the preparation phase was built on fictional patient personas, resulting in a representation of heterogeneous patient characteristics, needs, and potential treatment targets that shaped the treatment vision. During the design phase, input from end users via focus groups provided insights on the treatment content, design, and structure, with an emphasis on flexibility and person-centeredness. In the testing phase, 11 patients and 3 therapists participated, and findings confirmed that a behavioral approach was considered suitable for individuals with chronic pain. In addition, digital delivery of the intervention using a microsession format combined with regular contact between therapists and patients was seen as beneficial by end users as the modules were overall perceived as helpful, understandable, and enjoyable. End users rated their experiences using the intervention as good or excellent, indicating initial satisfaction with both the treatment and wider implementation procedure, such as the use of digital platforms for delivery as well as research-related informed consent and data collection.

Finally, end users provided suggestions for further improving the design and content, such as a text-to-speech function, additional content for specific needs such as insomnia, and aesthetic adjustments through images and videos.

### End-User Involvement: Complementary and Contradictory Views

#### Overview

Critically reflecting on the input provided by end users regarding the utility of the development process and the DAHLIA prototype 1.0 is important for outlining the next steps in the DAHLIA project, namely, the further treatment improvements, testing, and implementation [30]. While the existing literature does not provide specific information on the recommended level of involvement of end users [50], user engagement is generally encouraged [20,51] and is considered a cornerstone in health care innovation [52]. Although many studies have focused on a single end-user group, such as patients or therapists, Rosser and Eccleston [53] emphasize the utility of including health care providers alongside patients during the development of a novel treatment [54].

However, while combining views from different stakeholders may be beneficial, it can also result in challenges. In this study, end-user input varied, with both consensus and discrepancies between individuals and end-user groups regarding preferences in treatment design. For instance, some patients preferred group-based treatment, whereas others favored individual treatment as it provided opportunities to choose their own pace. Moreover, patients mentioned that digital delivery may be suitable for some but can be a barrier to engage for specific groups such as older adults with chronic pain, which is in line with research highlighting that the digitalization of health care systems may result in the exclusion especially of those who are older and of a lower socioeconomic status [55]. These differing views emerged during phase 1 (design).

Importantly, patients’ and therapists’ preferences regarding the mode of communication differed. While patients generally expressed a preference for video calls over phone calls, therapists viewed phone calls as similarly useful as video calls and suggested that patients should be able to choose. This finding is in line with results from a systematic review aimed at examining the attitudes toward video- and phone-based telehealth [56]. Even though some studies presented no significant differences between patients’ and health care providers’ attitudes toward the mode of communication [57], others preferred video calls, and only a small number preferred phone calls [58]. This lack of consensus in the field underlines the importance of flexibility and adapting the mode of communication to patients’, and potentially also therapists’, preferences and needs. In the testing phase, therapists and patients decided together how to communicate, and the number of phone calls and video meetings was nearly equal, with phone calls being slightly more preferred. Future studies can empirically compare modes of communication to explore, for instance, whether dropout rates or user experiences vary based on this treatment feature. This flexibility was provided in phase 2, pilot-testing, where therapists and patients decided together on the mode of communication, aiming to incorporate flexibility and personalization.

Examining treatment content and design, both patients and therapists perceived a need to condense the text reduce the risk of the treatment being (too) challenging for some patients and provided helpful suggestions, such as the use of bullet points, overviews, or summaries. In addition, they highlighted the importance of incorporating images into the intervention to illustrate information and a text-to-speech function to enhance accessibility. This feedback led to the incorporation of bullet points, summaries, images, and audio files in the full prototype. While a built-in text-to-speech function is not supported by the 1177 platform, we plan to include this feature in future iterations through an additional solution in collaboration with IT developers. Overall, during the development and adaptation of a digital treatment, researchers face the challenge to balance “ideal” and “feasible” ideas as some intervention components may require resources (ie, time, technical features, funding, and expertise) that are not available.

In the testing phase, end-user experiences also varied between individuals. While therapists’ ratings were rather similar, patients’ experiences showed larger differences. In the weekly evaluations, where patients evaluated each module, and in the exit interviews, where they evaluated the intervention in general, they provided ratings that spanned almost the entire scale (from 1 to 7), indicating that individual differences may influence how the treatment is perceived. This finding points to a need to balance the end-user voices with practical requirements during the development process to optimize feasibility. Overall, the structure of this study, initially from conceptual preparation to collaborative design and, finally, to testing, enabled a gradual integration of feedback, allowing for tailoring the intervention to end-user needs.

#### Heterogeneity of the Target Population: One Size Does Not Fit All

A key challenge in designing a digital intervention is meeting varying individual needs [59]. Therefore, for the preparation phase of development, patient personas of different age groups, comorbidities, and pain experiences were created as potential representatives of the target population, aiming to amplify patient perspectives early on. Although seemingly useful, The application of patient personas in designing novel pain treatments is not standardized [37]. Groos et al [60] have suggested that different types of patient personas can be insightful for researchers while deciding the next steps of the development process. In the DAHLIA project, experiences from the preparation phase suggest that patient personas can also be helpful in the continuous refinement of the treatment or in tailoring the treatment to different patient groups to initially consider important characteristics such as disabilities, socioeconomic status, language skills, or cultural background that may reflect varying needs.

The heterogeneity in the chronic pain population is well known [1], and during the testing phase, patients with various pain profiles, such as migraine, chronic fatigue syndrome, herniated disc, arthritis, fibromyalgia, complex regional pain syndrome, and hypermobile Ehlers-Danlos syndrome, provided valuable input.

Therapists suggested that treatment should be tailored according to the patients’ needs and highlighted the importance of providing inclusive and diverse examples as part of exercises within the treatment. In a consensus statement that aims to provide a practical guideline for researchers in the field of e–mental health, Seiferth et al [23] emphasized the necessity of considering the target group when deciding the structure (eg, exercises and division into modules) and the complexity of the content. Similarly, Evangelista et al [61] highlighted the significance of participatory involvement of those who might be considered minority groups during the design of an intervention to better determine and meet their needs.

To address the varied preferences and needs of people with chronic pain, the intervention was made flexible and adaptable in its content and structure. The intervention was delivered over a 6-week period through microsessions that participants could complete at their own pace. Participants were able to choose the mode of communication (eg, text, video call, and phone call) for the weekly contact with their therapists. The content and exercises were not specific to a single group with chronic pain (eg, patients on sick leave) but were instead developed with a transdiagnostic approach (based on acceptance and commitment therapy and CBT principles) to ensure that the content was relevant to a wider range of people with chronic pain. In future iterations of the DAHLIA intervention, we aim to incorporate optional add-on modules (eg, sleep-related content) tailored to specific needs.

### Strengths and Limitations

By involving both patients and therapists in the development of the initial treatment version, this study adopted a user-centered approach that adapts the intervention to end users’ needs and preferences. In addition, this study had a strong emphasis on empirical data and various methodologies, integrating qualitative feedback and quantitative ratings. Patient personas were used to create awareness of potential target populations and their diverse needs. The treatment was tested in heterogeneous samples, providing insights into applicability across various demographics. In addition, the integration of established frameworks such as the CFIR strengthened the design.

Several limitations need to be considered when interpreting the results. First, a larger sample of end users would have provided more information and potentially a more diverse group. There was a discrepancy between the number of participants involved in patient (n=5) and therapist (n=12) focus groups. The targeted sample size for patients could not be reached as interest was low, 2 patients dropped out, and the project period did not allow for further recruitment. Even though the number of dropouts was low in this study, the small number of noncompleters limited our ability to analyze potential differences in demographics or engagement. Future iterations with larger samples will allow for more robust dropout analyses. While more therapists than anticipated participated, the limited number of patients raised the risk that data saturation was not achieved, particularly given the goal of capturing a heterogeneous population. This remains a limitation, although the enrollment of 11 patients in the pilot test may have strengthened patient voices. Second, more female than male participants took part in the study. A total of 5 patients and 12 therapists took part in the focus groups, most of whom were female (n=4, 80% of the patients and n=9, 75% of the therapists). In the pilot phase, 11 patients and 3 therapists participated, and they were mostly female (n=9, 82% of the patients and n=3, 100% of the therapists), which is consistent with similar studies [26,62]. While this gender distribution aligns with the higher prevalence of chronic pain in women [63,64] and the female dominance in psychology and psychotherapy professions [65], this imbalance should be considered when interpreting the findings as it may have influenced the feedback and preferences on the intervention design and content. Future studies should include more male end users and explore potential gender differences in preferences and needs.

In addition, the narrow range of pain diagnoses may limit the generalizability of the results. While there was a diversity of pain diagnoses among patients in the pilot test, only 40% (2/5) of the focus group participants had received a specific diagnosis (Ehlers-Danlos syndrome; 3/5, 60% were undiagnosed or did not know the diagnosis). Although the inclusion criteria aimed to increase heterogeneity, this limited diagnostic representation may have constrained the variety of preferences and needs reflected and may result in an intervention that aligns more with the experiences of certain chronic pain populations. Previous research shows that people with different chronic pain conditions can vary in treatment expectations [66], highlighting the need for representativeness and inclusivity when tailoring interventions for different subgroups. Therefore, future iterations of the DAHLIA project should ensure that the intervention aligns with the needs of the broader pain population.

This limited diversity suggests that the findings may not fully represent a broader population, highlighting the need to examine inclusivity in future research. Incorporating diverse groups—considering gender identity, sexual orientation, age, race and ethnicity, diagnosis, and medical and psychiatric comorbidities—can provide broader end-user feedback. To address this limitation, future iterations of the DAHLIA project will implement a broader recruitment strategy by expanding outreach to hospitals and clinics across different regions of Sweden and leveraging social media and online platforms (eg, websites and podcasts). These efforts aim to increase sample size and enhance demographic diversity regarding gender, age, and pain diagnosis. In addition, while the intervention is currently available only in Swedish, it can be culturally adapted and delivered in additional languages to improve accessibility [67,68].

On the basis of the development and initial testing described in this paper, the next steps of the DAHLIA study will include further refinement building on the pilot results, followed by an iterative optimization study and a randomized controlled trial [30]. These upcoming phases will assess the DAHLIA intervention’s feasibility, acceptability, and implementation potential in a larger scale. The insights from this study will inform the future phases, aiming to ensure that the intervention aligns with user preferences and needs.

### Conclusions

This study aimed at presenting the user-centered development of a digital behavioral intervention for chronic pain, the DAHLIA prototype 1.0. The results illustrate the utility of patient personas when preparing, of focus groups when designing, and of end-user feedback when testing this new intervention. The findings indicated that the treatment holds promise and provided relevant end-user suggestions to guide further improvements.

## Data Availability

The datasets generated or analyzed during this study are not publicly available due to data privacy but are available from the corresponding author (AST) on reasonable request.

## Appendix

Multimedia Appendix 1 Guidance for the Reporting of Intervention Development checklist, a guideline for reporting intervention development studies, and Template for Intervention Description and Replication checklist.

Multimedia Appendix 2 One of the 3 patient personas used in the development phase of the DAHLIA project (Swedish name: Göran).

Multimedia Appendix 3 Semistructured focus group guide.

Multimedia Appendix 4 Consolidated Framework for Implementation Research construct names and their definitions from the “Innovation Characteristics” (refers to the “digital intervention”) domain applied in the user-centered development phase of the DAHLIA digital behavioral intervention for people with chronic pain in Sweden.

Multimedia Appendix 5 Semistructured exit interview guide to evaluate the general feasibility and acceptability of the treatment for patients.

Multimedia Appendix 6 Semistructured exit interview guide to evaluate the general feasibility and acceptability of the treatment for health care professionals.

Multimedia Appendix 7 Consolidated Framework for Implementation Research construct names and their definitions from the Implementation Process domain applied in the piloting of the DAHLIA digital behavioral intervention for people with chronic pain in Sweden.

Multimedia Appendix 8 Examples (screenshots) of the prototype of the DAHLIA digital behavioral intervention for people with chronic pain.

## Abbreviations

CBT: cognitive behavioral therapy
CFIR: Consolidated Framework for Implementation Research
DAHLIA: Digital Behavioural Health for Chronic Pain
GUIDED: Guidance for the Reporting of Intervention Development
REDCap: Research Electronic Data Capture
