# Supplementary material for: User-Driven Development of a Digital Behavioral Intervention for Chronic Pain: Multimethod Multiphase Study
Source: JMIR Form Res. 2025 Jul 8;9:e74064. doi: 10.2196/74064 (PMC12284454; doi:10.2196/74064)
Supplement: Multimedia Appendix 1 [file formative_v9i1e74064_app1.docx]

| *GUIDED checklist item description*  (Duncan et al., 2020) | Where in the manuscript this is described | Other notes |
| --- | --- | --- |
| 1. Report the context for which the intervention was developed. | *Introduction* (mention of chronic pain population, accessibility issues, the need for digital behavioral interventions) | See also DAHLIA study protocol (Bartels et al., 2022) |
| 2. Report the purpose of the intervention development process. | *Introduction,*  *2.1 Study Design* |  |
| 3. Report the target population for the intervention development process. | *2.1 Study design*,  *2.4.2 Patients and recruitment*, *2.5.1 Patients and recruitment,*  *3.1. Phase 0 - Preparation* |  |
| 4. Report how any published intervention development approach contributed to the development process. | *Introduction*,  *2.3.1 Theoretical frameworks and conceptual model* |  |
| 5. Report how evidence from different sources informed the  intervention development process. | Figure 1,  *2.3. Phase 0 – Preparation: Preliminary patient characteristics, needs and treatment targets,*  *2.4. Phase 1 - Designing: User-centered design of the digital intervention,*  *2.5. Phase 2 - Testing: Piloting the digital intervention* |  |
| 6. Report how/if published theory informed the intervention  development process. | *Introduction*,  *2.3.1 Theoretical frameworks and conceptual model* |  |
| 7. Report any use of components from an existing intervention  in the current intervention development process. | *2.3.3 Intervention structure for prototype version 0.0* |  |
| 8. Report any guiding principles, people or factors that were prioritised when making decisions during the intervention development process. | *2.3.2 Patient Personas,*  *2.4.1. End-user focus groups,*  *2.5. Phase 2 - Testing: Piloting the digital intervention,*  *3.3.4. End-user suggestions for further treatment improvements* |  |
| 9. Report how stakeholders contributed to the intervention  development process. | *2.4.2. Participants and recruitment,*  *2.5.1 Participants and recruitment* |  |
| 10. Report how the intervention changed in content and format from the start of the intervention development process. | *3.3.4. End-user suggestions for further treatment improvements,*  Figure 5 |  |
| 11. Report any changes to interventions required or likely to be required for subgroups. | *3.2.2.3 Adaptability of the treatment,*  *4.2 Heterogeneity of the target population: One size does not fit all,*  *Strengths and Limitations* |  |
| 12. Report important uncertainties at the end of the intervention development process. | *4.1 End-users involvement: Complementary and contradictory views,*  *4.2 Heterogeneity of the target population: One size does not fit all,*  *Strengths and Limitations* |  |
| 13. Follow TIDieR guidance when describing the developed intervention. | Detailed description of the intervention is provided in the TIDieR checklist (submitted as a supplementary file) |  |
| 14. Report the intervention development process in an open access format. | This paper is reported in an open access format in accordance with the checklist. |  |

| *TIDieR checklist item (with item numbers)*  (Hoffmann et al., 2014) | Description |
| --- | --- |
| 1. Brief name:  Provide the name or a phrase that describes the intervention. | The DAHLIA digital behavioral intervention for people with chronic pain |
| 2. Why:  Describe any rationale, theory, or goal of the elements essential to the intervention | The DAHLIA intervention was developed to address the needs in chronic pain by offering an evidence-based, user-centered, and flexible digital behavioral treatment. |
| 3. What:  *Materials*: Describe any physical or informational materials used in the intervention, including those provided to participants or used in intervention delivery or in training of intervention providers. Provide information on where the materials can be accessed (e.g. online appendix, URL)  4.What: | The intervention included six weekly modules delivered via a digital platform (1177.se). |
| *Procedure:* Describe each of the procedures, activities, and/or processes used in the intervention, including any enabling or support activities. | Participants accessed one module per week over six weeks. They were instructed to review the materials, complete weekly exercises, and reflect on their values and goals. Participants had weekly contact with their assigned therapist after completion of each module. |
| 5. Who provided:  For each category of intervention provider (e.g. psychologist, nursing assistant), describe their expertise, background and any specific training given. | The DAHLIA intervention is self-guided, patients had weekly contact with a therapist. In pilot testing (Phase 2), three female licensed psychologists delivered the treatment with 21.7 years of experience with providing psychological therapy, (SD: 13.31, range: 7-33), 18.3 years of experience working specifically with people with chronic pain (SD: 14.6, range 2-30), and 12 years of experience of using ACT (SD: 9.84, range 1-20) (see section 3.3.1 and |
| 6. How:  Describe the modes of delivery (e.g. face-to-face or by some other mechanism, such as internet or telephone) of the intervention and whether it was provided individually or in a group. | The DAHLIA intervention is self-guided and delivered via a secure web platform accessible by computer and mobile device. Patients had weekly contact with their therapists, where patients and therapists together chose the mode of communication (phone or video call). |
| 7. Where:  Describe the type(s) of location(s) where the intervention occurred, including any necessary infrastructure or relevant features. | The DAHLIA intervention delivered digitally; participants accessed it remotely at at their own convenience using personal devices (e.g., computer, tablet). |
| 8. When and how much:  Describe the number of times the intervention was delivered and over what period of time including the number of sessions, their schedule, and their duration, intensity or dose. | The DAHLIA intervention consisted of six modules, with one module released each week, including weekly contact with a therapist where patients and therapists together chose the mode of communication (phone or video call) as well as two booster sessions (after 2- and 4-months). Each module included four micro-sessions, resulting in a total of 24 micro-sessions. |
| 9. Tailoring:  If the intervention was planned to be personalised, titrated or adapted, then describe what, why, when, and how. | The DAHLIA intervention adopts a user-centered approach that adapts the intervention to end-users' needs and preferences |
| 10. Modifications:  If the intervention was modified during the course of the study, describe the changes (what, why, when, and how). | Changes were made to the prototype based on feedback from focus groups during piloting. |
| 11. How well:  *Planned*: If intervention adherence or fidelity was assessed, describe how and by whom, and if any strategies were used to maintain or improve fidelity, describe them.  12. How well: | N/A |
| *Actual:* If intervention adherence or fidelity was assessed, describe the extent to which the intervention was delivered as planned. | N/A |

1. Duncan, E., et al., Guidance for reporting intervention development studies in health research (GUIDED): an evidence-based consensus study. BMJ open, 2020. 10(4): p. e033516

2. Hoffmann, T.C., et al., Better reporting of interventions: template for intervention description and replication (TIDieR) checklist and guide. Bmj, 2014. 348.
