# Supplementary figures and images for: User-Driven Development of a Digital Behavioral Intervention for Chronic Pain: Multimethod Multiphase Study
Source: JMIR Form Res. 2025 Jul 8;9:e74064. doi: 10.2196/74064 (PMC12284454; doi:10.2196/74064)

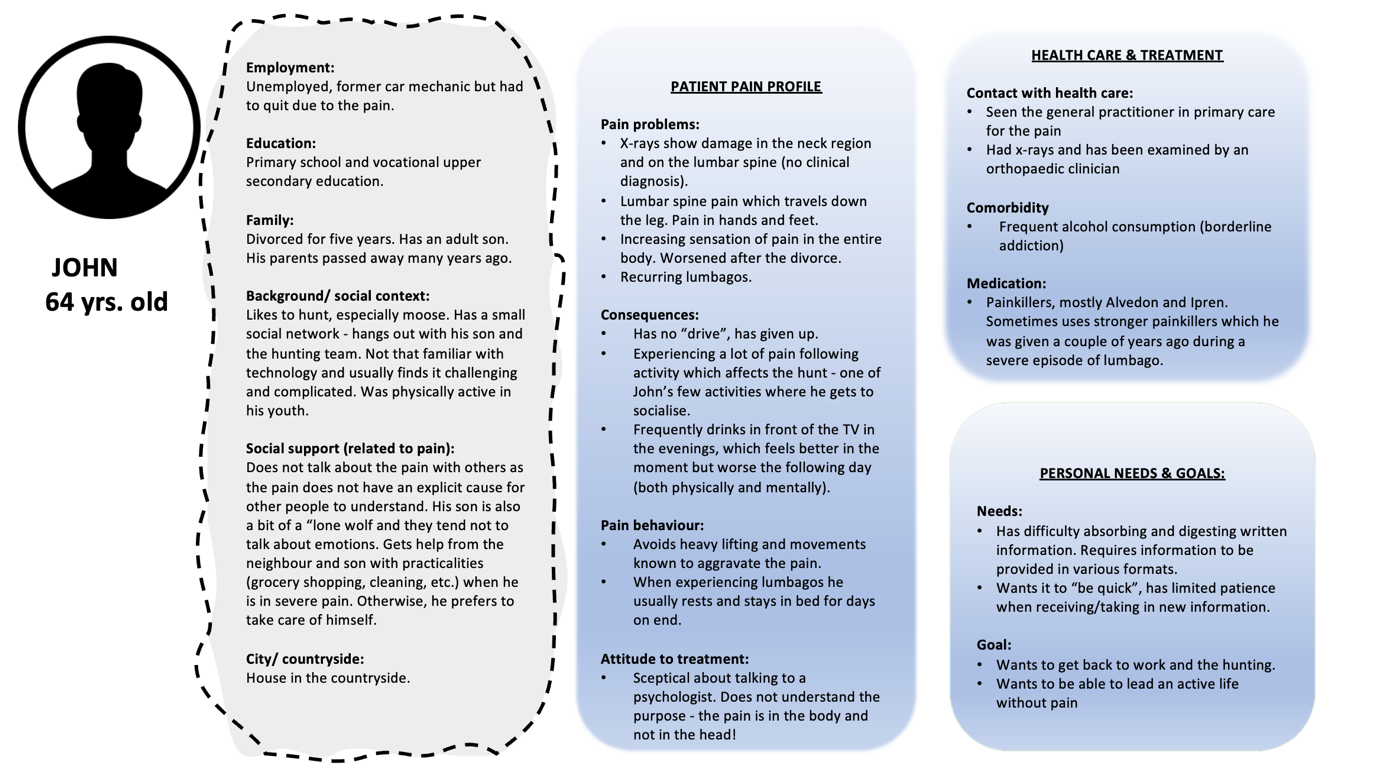

Supplement: Multimedia Appendix 2 [file formative_v9i1e74064_app2.docx]

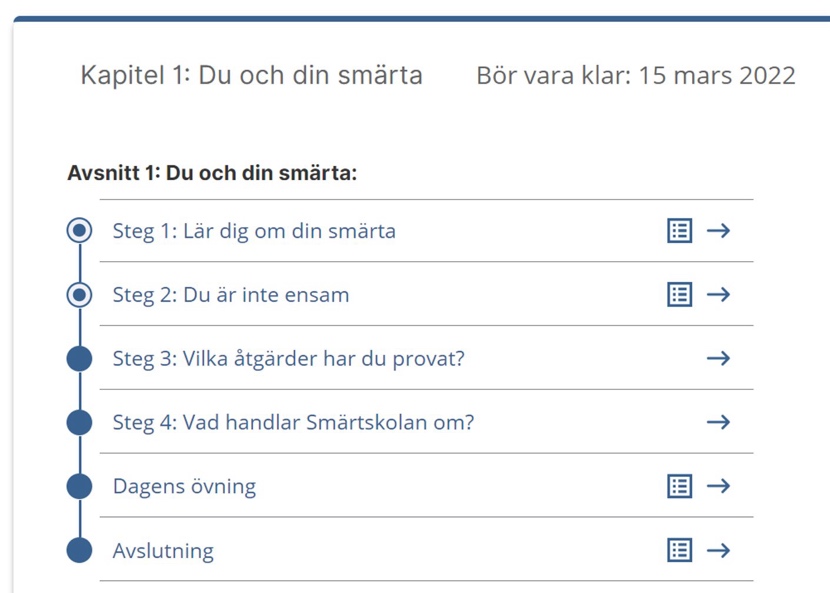


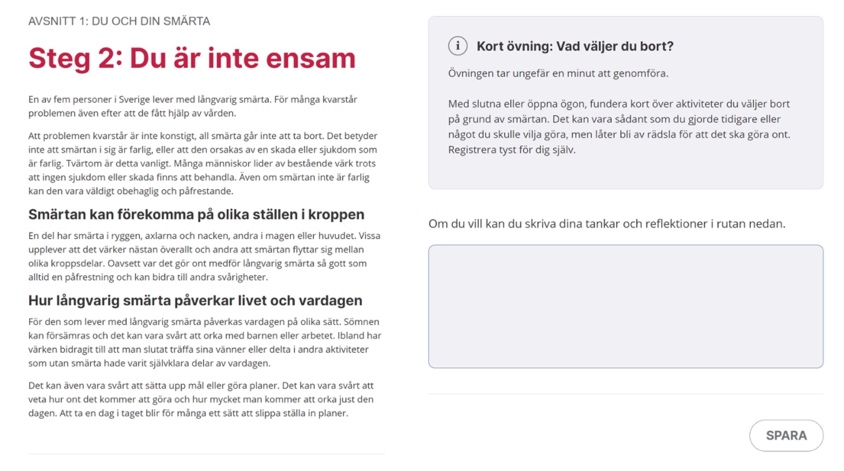

Supplement: Multimedia Appendix 8 [file formative_v9i1e74064_app8.docx]
