# Supplementary material for: User-Driven Development of a Digital Behavioral Intervention for Chronic Pain: Multimethod Multiphase Study
Source: JMIR Form Res. 2025 Jul 8;9:e74064. doi: 10.2196/74064 (PMC12284454; doi:10.2196/74064)
Supplement: Multimedia Appendix 3 [file formative_v9i1e74064_app3.docx]

**Semi-structured focus group guide**

6-8 participants per focus group

FOR PATIENTS (*2 focus groups; heterogenic in terms of age, gender, pain condition, pain history, etc.*):

1. General introduction, informed consent, collect sociodemographic details (10min.)
2. Short introduction round (10min.)

1. Core question 1: **Living with chronic pain** (30min.)

It would be amazing to have a magic pill to just take all the pain away, so you could live without it. But unfortunately, we don’t have that magic pill. Instead, we want to help you and other people with chronic pain to find a way to live well with the pain. (*Presentation on definition of health (Huber et al., 2011): ability to adapt and self-manage physical, mental and social aspects of health, and examples*).

1. Based on this definition of health, can you describe your own health needs? Which (aspects of your) needs are currently unmet?
2. In which moments of your life do you feel happiest/ most engaged/ most satisfied?
3. What helps you to engage in these ‘happy moments’?
4. What are barriers to engage in these ‘happy moments’?
5. What would you need to engage in these moments more often?

BREAK 10 Min.

1. Core question 2: **The DAHLIA treatment**

Presentation of the proposed treatment, aim, design, theoretical background, and examples of exercises (10min); following a discussion (30min)

1. What do you think of this treatment? What do you like, what do you dislike? (Please reflect on (1) design, (2) set-up, (3) content, (4) other (e.g., terminology: treatment, intervention, program; patient vs. person))
2. How feasible would it be to do this treatment?
3. Do you think this treatment meets you needs?
4. Is there anything else you would like to add?

FOR HEALTH CARE PROFESSIONALS (*1 focus group, psychologists/ psychotherapists trained in cognitive-behavioural therapy;* *heterogenic in terms of age, gender, cultural background)*:

1. General introduction, informed consent, collect sociodemographic details (10min.)
2. Short introduction round (10min.)

1. Core question 1: **Supporting people with chronic pain** (30min)

People with chronic pain have complex needs and treatment has to meet these needs. We are interested in your experiences in what works well to improve the overall health and well-being of patients with chronic pain. (*Presentation on definition of health (Huber et al., 2011): ability to adapt and self-manage physical, mental and social aspects of health, and examples*).

1. Which (aspects of) your patient’s health needs are unmet? What is needed to support chronic pain patients in the best way?
2. What barriers and facilitators to deliver support to chronic pain patients do you face? Please reflect on elements related to the patient, treatment options, and the health care in general.

BREAK 10 Min.

1. Core question 2: **The DAHLIA treatment**

Presentation of the proposed treatment, aim, design, theoretical background, and examples of exercises (10min); following a discussion (30min)

1. What do you think of this treatment? What do you like, what do you not like? (Please reflect on (1) design, (2) set-up, (3) content, (4) other (e.g., terminology: treatment, intervention, program; patient vs. person))
2. How feasible would it be for you to deliver this treatment?
3. Does the treatment meet the needs of the patients with chronic pain?
4. Is there anything else you would like to add?
