# Supplementary material for: User-Driven Development of a Digital Behavioral Intervention for Chronic Pain: Multimethod Multiphase Study
Source: JMIR Form Res. 2025 Jul 8;9:e74064. doi: 10.2196/74064 (PMC12284454; doi:10.2196/74064)
Supplement: Multimedia Appendix 4 [file formative_v9i1e74064_app4.docx]

| **CFIR Domain** | **Construct name** | **Construct definition** |
| --- | --- | --- |
|  |  |  |
| Innovation:  *The DAHLIA treatment prototype* | Evidence-Base | The effectiveness of the intervention is supported by robust evidence |
|  | Relative Advantage | The intervention is better than other available innovations or current practice |
|  | Adaptability | The intervention can be modified, tailored, or refined to fit local context or needs |
|  | Complexity | The intervention is complicated, which may be reflected by its scope and/or the nature and number of connections and steps |
|  | Design | The intervention is well designed and packaged, including how it is assembled, bundled, and presented |
