# Supplementary material for: User-Driven Development of a Digital Behavioral Intervention for Chronic Pain: Multimethod Multiphase Study
Source: JMIR Form Res. 2025 Jul 8;9:e74064. doi: 10.2196/74064 (PMC12284454; doi:10.2196/74064)
Supplement: Multimedia Appendix 5 [file formative_v9i1e74064_app5.docx]

| **Topics** | **Questions** | **Answering scores** | **Open question** |
| --- | --- | --- | --- |
| You recently completed the 6-week treatment. Your experiences help us to improve the treatment. Thank you for taking the time to provide us with your input. First, we would like to ask you to reflect on and rate the **past weeks and treatment** in general. | | | |
| General | 1.Would you consider the past 6 weeks “ordinary”? | 7-points scale: from  1=’not at all’ to  7= ‘very much’ | Please elaborate if possible |
|  | 2.Did anything unusual occur during the treatment period? |  |  |
|  | 3.Did the online treatment interfere with your daily routines (work or other things)? |  |  |
|  | 4.Would you recommend this online treatment to a friend with a similar condition? |  |  |
|  | 5.Did you experience the online treatment as helpful overall? |  |  |
|  | 6.Did you experience the online treatment as meaningful overall? |  |  |
| Secondly, we would like to ask you to reflect on and rate the **sessions, overall treatment, and 1177 online environment**. | | | |
| Micro-sessions/ online environment | 7.Was the written material understandable? | 7-points scale: from  1=’not at all’ to  7= ‘very much’ | Please elaborate if possible |
|  | 8.The treatment was delivered using a digital platform on 1177. Was the information about the digital 1177 platform clear? |  |  |
|  | 9.Was it easy to navigate the digital 1177 platform? |  |  |
|  | 10.Could you easily read the text in the treatment (i.e., in terms of font size and formatting)? |  |  |
|  | 11.Was the number of sessions per week (4 sessions) adequate? (Follow-up if needed: Would you prefer more/less (open question)? |  |  |
|  | 12.Was the total number of sessions adequate? (Follow-up if needed: Would you prefer more/less (open question)? |  |  |
|  | 13.Was the time needed to complete the sessions acceptable? |  |  |
|  | 14.Did you experience any technical problems using 1177? |  |  |
|  | 15.Did micro-sessions influence your behavior in everyday life? |  |  |
|  | 16.Did micro-sessions influence your emotions? |  |  |
|  | 17.Did micro-sessions influence your thoughts? |  |  |
| Third, we would like to ask you to reflect and rate the communication with your health care professional. | | | |
| Messenger function/ health care professional | 18.Which communication tool(s) (e.g., messenger function, phone call, video call) was used when communicating with the health care professional? (Multiple choice) | 7-points scale: from  1=’not at all’ to  7= ‘very much’ | Please elaborate if possible |
|  | 19.Did you experience communicating with your health care professional as helpful overall? |  |  |
|  | 20.Was it easy to schedule meetings with your health care professional? |  |  |
|  | 21.Did you experience communicating with your health care professional as motivating? |  |  |
|  | 22.Did you feel supported by your health care professional? |  |  |
| 33.In which way did the treatment improve your well-being? | | | Please elaborate |
| 34.Is there anything else you would like to add? | | | Free text |
