# Supplementary material for: User-Driven Development of a Digital Behavioral Intervention for Chronic Pain: Multimethod Multiphase Study
Source: JMIR Form Res. 2025 Jul 8;9:e74064. doi: 10.2196/74064 (PMC12284454; doi:10.2196/74064)
Supplement: Multimedia Appendix 6 [file formative_v9i1e74064_app6.docx]

| **Topics** | **Questions** | **Answering scores** | **Open question** |
| --- | --- | --- | --- |
| You recently provided the 6-week treatment to patients with chronic pain. For us, it is very important to hear how you experienced it so that we can improve the content, design, and other aspects further. Thank you for taking the time to provide us with your input. | | | |
|  | 1.To how many patients did you deliver the digital intervention to? | (add number) |  |
| General | 2.Did you experience any technical problems using 1177/SOB? | 7-points scale:  from  1=’not at all’ to  7= ‘very much’ | Please elaborate if possible |
|  | 3.Was it easy to navigate 1177/SOB? |  |  |
|  | 4.Was the online treatment overall beneficial for your patients? |  |  |
|  | 5.Did you feel prepared to deliver this treatment? |  |  |
|  | 6.Was the support for delivering the intervention (e.g., training, technical guidance when issues arose, supervision) sufficient? |  |  |
|  | 7.How satisfied are you with the intervention overall? |  |  |
|  | 8.Was the frequency of communication with the patient acceptable? |  |  |
|  | 9.Was the time per interaction (e.g., phone call) acceptable? |  |  |
|  | 10.Was the overall time investment to deliver the online treatment acceptable? |  |  |
|  | 11.Did delivering this treatment online save you time? |  |  |
|  | 12.Was the online treatment delivered as intended? |  |  |
|  | 13.Would you deliver the intervention again in the future? |  |  |
|  | 14.Would you recommend the intervention to a colleague? |  |  |
|  | 15.What facilitated you to deliver the intervention? | Open question |  |
|  | 16.What hindered you in delivering the intervention? | Open question |  |
|  | 17.What aspects of the intervention need improvements? | Open question |  |
| 18.Is there anything else you would like to add? | | | Free text |
