# Supplementary material for: User-Driven Development of a Digital Behavioral Intervention for Chronic Pain: Multimethod Multiphase Study
Source: JMIR Form Res. 2025 Jul 8;9:e74064. doi: 10.2196/74064 (PMC12284454; doi:10.2196/74064)
Supplement: Multimedia Appendix 7 [file formative_v9i1e74064_app7.docx]

| **CFIR Domain** | **Construct name** | **Construct definition** |
| --- | --- | --- |
| Implementation Process    *The activities and strategies used to implement the intervention.* | Reflecting & Evaluating: Intervention | 1. Intervention: Qualitative and quantitative information that is collected to assess the **success of the intervention** |
|  | Reflecting & Evaluating: Implementation | 2. Implementation: Qualitative and quantitative information that is collected to assess the **success of the implementation** |
